# Single-cell transcriptomic analysis of normal and pathological tissues from the same patient uncovers colon cancer progression

**DOI:** 10.1186/s13578-023-01002-w

**Published:** 2023-03-21

**Authors:** Ruifang Sun, Yang Yang, Weidong Lü, Yanqi Yang, Yulong Li, Zhigang Liu, Dongmei Diao, Yang Wang, Su’e Chang, Mengnan Lu, Qiuyu Jiang, Bingling Dai, Xiaobin Ma, Chang’an Zhao, Moqi Lü, Juan Zhang, Caixia Ding, Na Li, Jian Zhang, Zhengtao Xiao, Dangxia Zhou, Chen Huang

**Affiliations:** 1 Department of Oncology Surgery, The First Affiliated Hospital of Xi’an Jiaotong University, 277 Yanta West Road, Xi’an, Shaanxi People’s Republic of China; 2 Department of Pathology, School of Basic Medical Sciences, Health Science Center, Xi’an Jiaotong University, 76 Yanta West Road, Xi’an, Shaanxi People’s Republic of China; 3 School of Public Health, Shaanxi University of Chinese Medicine, Middle Section of Century Avenue, Xianyang, Shaanxi People’s Republic of China; 4 Department of Thoracic Surgery, Shaanxi Provincial Tumor Hospital, Xi’an Jiaotong University, 309 Yanta West Road, Xi’an, Shaanxi People’s Republic of China; 5 Department of Gastroenterology, Shaanxi Provincial People’s Hospital, 256 Youyi West Road, Xi’an, Shaanxi People’s Republic of China; 6 Department of Orthopedics, The Second Affiliated Hospital of Xi’an Jiaotong University, 157 Xiwu Road, Xi’an, Shaanxi People’s Republic of China; 7 Department of Pediatrics, The Second Affiliated Hospital of Xi’an Jiaotong University, 157 Xiwu Road, Xi’an, Shaanxi People’s Republic of China; 8 Department of Cell Biology and Genetics, School of Basic Medical Sciences, Health Science Center, Xi’an Jiaotong University, 76 Yanta West Road, Xi’an, Shaanxi People’s Republic of China; 9 School of Pharmacy, Health Science Center, Xi’an Jiaotong University, 76 Yanta West Road, Xi’an, Shaanxi People’s Republic of China; 10 Department of Oncology, The Second Affiliated Hospital of Xi’an Jiaotong University, 157 Xiwu Road, Xi’an, Shaanxi People’s Republic of China; 11 Department of Pathology, Shaanxi Provincial Tumor Hospital, Xi’an Jiaotong University, 309 Yanta West Road, Xi’an, Shaanxi People’s Republic of China; 12 School of Pharmacy, Xinxiang Medical University, 601 Jinsui Avenue, Xinxiang, Henan People’s Republic of China; 13 Department of Biochemistry and Molecular Biology, School of Basic Medical Sciences, Health Science Center, Xi’an Jiaotong University, 76 Yanta West Road, Xi’an, Shaanxi People’s Republic of China

**Keywords:** Single cell, Colon adenoma, Colon cancer, Cancer development

## Abstract

The aim of the present study was to elucidate the evolutionary trajectory of colon cells from normal colon mucosa, to adenoma, then to carcinoma in the same microenvironment. Normal colon, adenoma and carcinoma tissues from the same patient were analyzed by single-cell sequencing, which perfectly simulated the process of time-dependent colon cancer due to the same microenvironment. A total of 22 cell types were identified. Results suggest the presence of dominant clones of same cells including C2 goblet cell, epithelial cell subtype 1 (Epi1), enterocyte cell subset 0 (Entero0), and Entero5 in carcinoma. Epi1 and Entero0 were Co-enriched in antibacterial and IL-17 signaling, Entero5 was enriched in immune response and mucin-type O-glycan biosynthesis. We discovered new colon cancer related genes including AC007952.4, NEK8, CHRM3, ANO7, B3GNT6, NEURL1, ODC1 and KCNMA1. The function of TBC1D4, LTB, C2CD4A, AND GBP4/5 in T cells needs to be clarified. We used colon samples from the same person, which provide new information for colon cancer therapy.

**Supplementary Information:**

The online version contains supplementary material available at 10.1186/s13578-023-01002-w.

## Highlights

- We performed single-cell transcriptome sequencing in one case carrying adenoma and carcinoma, and explored process of colon cancer progression.
- Results suggest the presence of dominant clones of same cells including C2 goblet cell, epithelial cell subtype 1 (Epi1), enterocyte cell subset 0 (Entero0), and Entero5 in colon carcinoma
- We discovered new colon cancer related genes including AC007952.4, NEK8, CHRM3, ANO7, B3GNT6, NEURL1, ODC1 and KCNMA1.
- The function of TBC1D4, LTB, C2CD4A, AND GBP4/5 in T cells needs to be clarified.

**Supplementary Information:**

The online version contains supplementary material available at 10.1186/s13578-023-01002-w.

## Introduction

The occurrence and development of colon cancer is a multi-stage process from adenoma to carcinoma. Colon cancer is an ideal model to study the multi-stages of carcinogenesis [1]. Adenomatous polyps are definite precancerous lesions [2]. Clinical research showed that the adenoma detection rate was inversely associated with the risks of interval colorectal cancer (CRC), advanced-stage interval cancer, and fatal interval cancer [3, 4].

Single-cell RNA sequencing (scRNA-seq) is a powerful analysis tool to reveal the gene expression state in a single cell. Research on colon cancer by scRNA-seq has been partially carried out. CRC showed obvious heterogeneity in single-cell level [5]. Researchers have performed scRNA-seq analyses on immune and stromal populations from patients with CRC and identified specific macrophage and conventional dendritic cell subsets as key mediators of cellular cross-talk in the tumor microenvironment [6]. Li et al. [7] performed scRNA-seq on the tumors and normal mucosa of patients with CRC, developed a new clustering method called reference component analysis.

Existing scRNA-seq studies of colon cancer have limitations, that is, samples were collected from different patients, and few works collected continuously different pathological stages of colon cancer in the same patient. The tumor microenvironment also varies indifferent patients, and mixed-microenvironment cells must mask this difference. In this study, we obtained the normal colon, adenoma, and carcinoma tissues from the same patient which carries adenoma and carcinoma at the same time and explored via scRNA-seq. We analyzed the overall evolution during transition from normal colon to adenoma and to carcinoma and the cell numbers and expression change patterns of epithelial cells, enterocyte cells, etc. Results describe the overall situation of transcriptome map in single-cell level and reflect the gradual change in colon cancer, which is of great significance to clarify dynamically the occurrence of colon cancer and patient’s individualized medical treatment.

## Materials and methods

### Specimen collection

The normal tissue, adenoma tissue, and colon cancer tissue of the same patient were collected. The patient had not received chemotherapy or radiotherapy before operation. Clinical information was obtained from the patient’s medical record. Patient’s informed consent was obtained. The study was approved by the ethics committee of Xi’an Jiaotong University.

### Library construction and sequencing

The prepared single-cell suspension and the antibody solution with the sample label were incubated. The sample was loaded into the U-card chip of BD Rhapsody (BD, USA), the magnetic beads were spread, and the excess beads were washed off. After standing for a period of time, cell lysis was conducted to combine free mRNA with the magnetic beads. The magnetic beads carrying the mRNA of single cells were recovered, reverse transcribed, and used to synthesize cDNA. After the completion of cDNA amplification, the enzyme sections were segmented, end repaired, added with A, and spliced. The amplified cDNA and library were evaluated by Agilent 4200. Finally, the sequencing was completed by pe150 strategy in Novaseq 6000 (Illumina, USA).

### Single-cell data processing

A single-cell software suite was used to process the raw data. FastQC software was used to evaluate the quality of the raw sequencing data, and FASTX-Toolkit was used to control the quality of fastq sequence. Seurat 3.0 (R package) was used for clustering. After filtering out cells with gene number < 200, gene number ranked in the top 1% or a mitochondrial gene ratio of > 25%. The dimension was reduced by principal component analysis (PCA), and the data were visualized by TSNE or UMAP.

### Copy number variation analysis

InferCNV package (https://github.com/broadinstitute/infercnv, v1.3.3) was used to infer the profiles of copy number variations based on the average expression of large group of genes in each chromosome. Single cell RNA-seq data of the normal tissue was served as the reference for CNV calling. Genes were arranged based on their chromosome coordinates. CNV for each cell was estimated by applying a moving average window with a length of 101 within each chromosome. Single cells from carcinoma tissue were clustered into three groups using the hierarchical clustering method. The cluster whose CNV pattern differs from that of reference cells and meanwhile displays high-frequency copy number alterations was assigned as cancer cells.

### Cluster marker recognition, cell type annotation and pseudotime analysis

SingleR was used to analyze cell type annotation. SingleR (https://bioconductor.org/packages/devel/bioc/html/SingleR.html) was used for unbiased cell-type recognition from single-cell RNA sequencing data by leveraging the reference transcriptomic datasets of pure cell types to infer the origin of each single cell independently. Human Cell Landscape reference datasets were used.

### Gene set variation analysis (GSVA)

Using hallmark gene sets in MsigDB as the gene set, we applied the standard settings in GSVA software to assign the estimated value of pathway activity to a single cell. We compared the activity score of each cell with the Limma software package and then evaluated the differential activity of pathways between cell subsets. The differential activity of the pathways of each identified cluster was calculated.

### Enrichment analysis

KEGG enrichment and DISEASE enrichment (human only) of cluster markers were performed with KOBAS software and Benjamini–Hochberg multiple testing adjustment by using the top 20 marker genes of the cluster. The results were visualized using R package.

### Gene expression profiling interactive analysis (GEPIA) analysis

GEPIA software can provide rapid and customizable analysis using TCGA (The Cancer Genome Atlas, https://www.cancer.gov/about-nci/organization/ccg/research/structural-genomics/tcga) and GTEX data. We used GEPIA to detect the expression of (selected genes) in colon cancer and its relationship to the prognosis of colon cancer.

### Hematoxylin–eosin staining (HE)

Paraffin sections of normal colon, adenoma, and carcinoma tissues were prepared. The paraffin specimens were treated with xylene and gradient ethanol for dewaxing and hydration. Hematoxylin semen and subsequent 1% hydrochloric acid ethanol were added. Finally, neutral gum sealing was performed. Two pathologists determined the diagnosis independently.

### Immunohistochemistry (IHC)

Some IHC results were obtained from The HPA (The Human Protein Atlas, https://www.proteinatlas.org/). Some IHC were performed by our lab. In brief, the paraffin sections were deparaffinized with xylene and hydrated using an alcohol gradient. Endogenous-peroxidase-blocking and antigen retrieval were conducted sequentially. The sections were then incubated with primary antibody followed by secondary antibody conjugated with horseradish peroxidase. Detection was conducted by 3,3′-diaminobenzidine (DAB) and hematoxylin. The score of immunostaining was evaluated by two independent pathologists [8, 9].

### Statistical analysis

R and SPSS 23.0 statistical software were used for data analysis. All data are presented as mean ± SD. Student’s t-test or one-way ANOVA was used to evaluate differences among groups. Data were considered to be statistically significant when P < 0. 05.

## Results

### Normal colon, adenoma, and carcinoma of the same patient via scRNA-seq

**Fig. 1:**
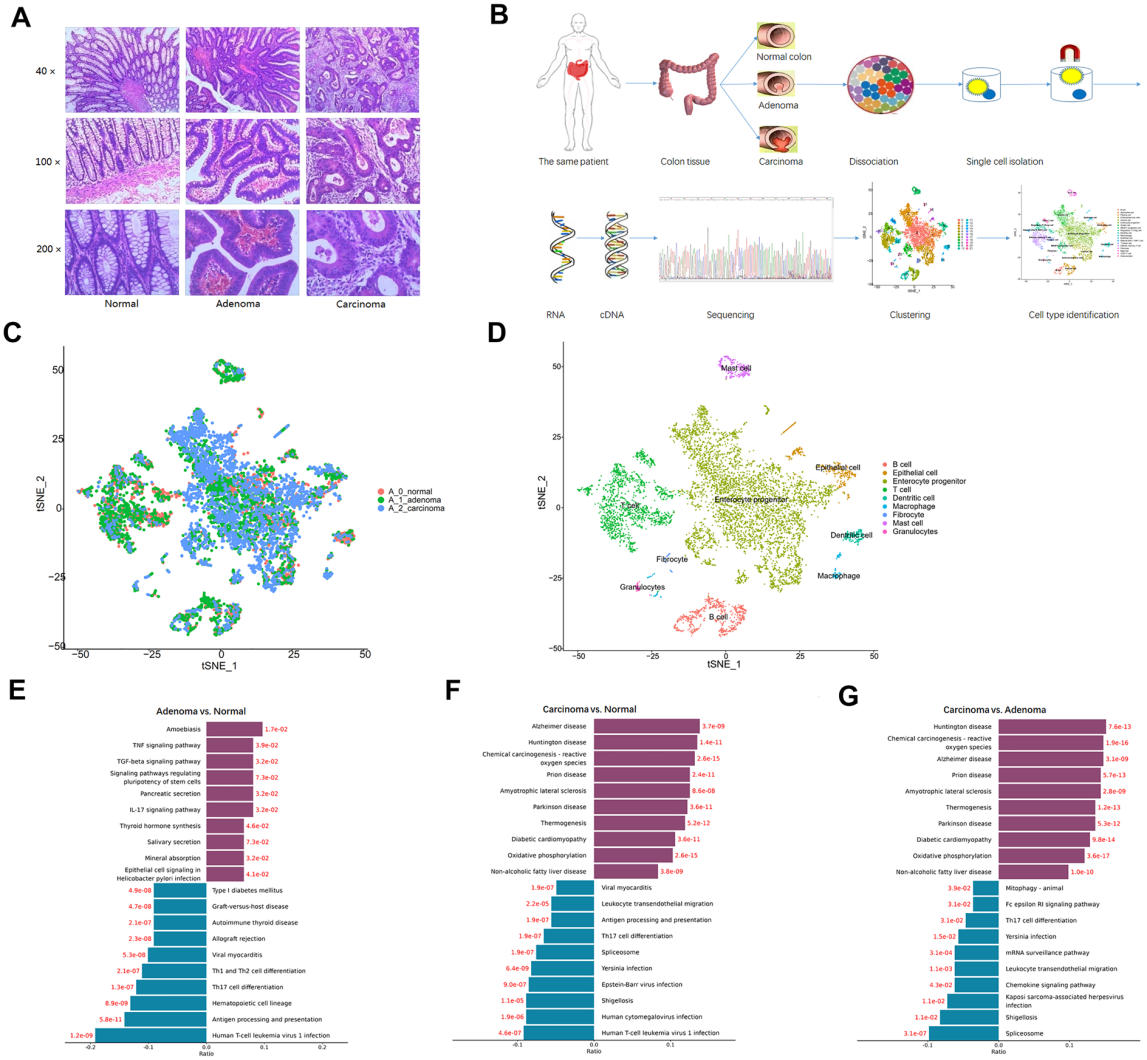
Experimental design of single RNA-seq on 3 staged colonic lesions in the same patient. **A**. Hematoxylin–eosin (H&E) staining of the tissues with different stages of colonic lesions including normal mucosa, colonic adenoma and carcinoma in the same patient. **B**. Overview of the experimental design of single cell RNA-seq. Pathological lesions of the colonic tissue including normal mucosa, adenoma and carcinoma were dissected and digested into single-cell suspensions for further separation, followed by library construction, sequencing, clustering and cell identification. **C**. tSNE plot of all the cells based on three different samples. **D**. tSNE plot of all the cells based on different cell types. **E**. KEGG enrichment of Top signal pathway in adenoma tissue compare to normal tissue, the blue bar and red bar show the down-regulated and up-regulated pathway, separately. **F**. KEGG enrichment of Top signal pathway in carcinoma tissue compare to normal tissue, the blue bar and red bar show the down-regulated and up-regulated pathway, separately. **G** KEGG enrichment of Top signal pathway in carcinoma tissue compare to adenoma tissue, the blue bar and red bar show the down-regulated and up-regulated pathway, separately

We performed a time sequential single-cell transcriptome profile analysis of colonic lesions at three different pathological stages including colon tissue, adenoma tissue, and carcinoma tissue (Fig. 1A, B). The sample diagnosis was conducted by Hematoxylin–eosin (HE) staining (Fig. 1A). A total of 17,397 cells were obtained. The results of the tSNE test showed the cell population distribution composed of cells from different sample sources (Fig. 1C). According to the expression of canonical markers and T-distribution random neighbor embedding (tSNE), we divided the cells into T cells, B cells, macrophages, dendritic cells, mast cells (and other immune cells), as well as enterocyte cells, epithelial cell, granulocytes cells, fibrocyte cells (and other non-immune cells) (Fig. 1D). KEGG analysis showed, compared with normal samples, carcinoma and adenoma tissues were preferentially enriched and suppressed the pathway of Th17 cell differentiation (Fig. 1E,F, G).

### Cell annotation and number composition ratio

**Fig. 2:**
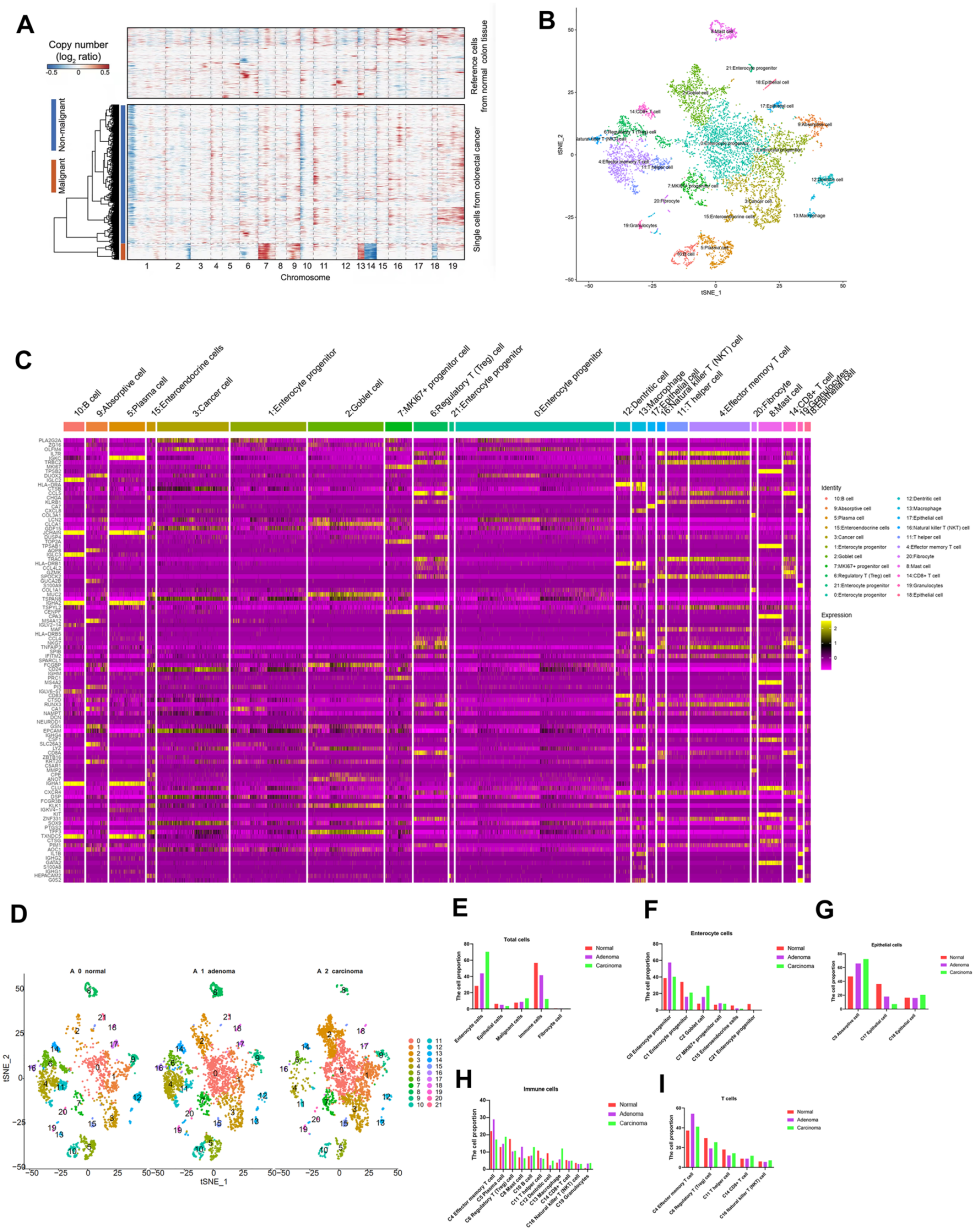
Distinct cell populations and their expression signatures. **A**. CNV was performed to identify the colorectal cancer cells. **B**. tSNE plot of all the cells based on different cell types and clusters. **C**. tSNE plot of all the cells based on different stages and clusters. **C**. The marker genes in each cell type were presented. **D**. The stacked histogram shows the 22 different cell cluster compositions in 3 pathological stages. **E**. The cell proportions of enterocyte cells, epithelial cells, malignant cells, immune cells, fibrocyte cells comparing to total cells. **F**. The cell proportions of subcluster-enterocyte cells comparing to enterocyte cells. **G**. The cell proportions of subcluster-epithelial cells comparing to epithelial cells. **H**. The cell proportions of subcluster-immune cells comparing to immune cells. **I**. The cell proportions of subcluster-T cells comparing to total T cells

Colon cancer cells were identified by using copy number variation analysis, and 22 groups were identified (Fig. 2A, B). The marker genes of the different cell types were presented in Fig. 2C, Additional file 2: Table S1. We stratified 22 groups of cells from three samples (Fig. 2D) and counted the cell proportion (Fig. 2E–I, Additional file 2: Table S2). Firstly, we separate these cells into large clusters, including enterocyte cells, epithelial cells, malignant cells, immune cells, fibrocyte cells. The results showed that in carcinoma tissues, the proportion of enterocyte cells and malignant cells were increased, while the immune cells were decreased. Secondly, we performed the subclustering analysis. In enterocyte cells, the proportion of C2 goblet cells were increased in carcinoma tissues, and proportion of enteroendocrine cells were decreased. In epithelial cells, the proportion of C9 absorptive cells were increased in carcinoma tissues. In immune cells, the proportion of C5 plasma cells, C10 B cells, C13 macrophage cells, C19 granulocyte cells were increased in carcinoma tissues. In T cells, the proportion of C4 effector memory T cells, C14 CD8 + T cells, C16 natural killer T (NKT) cells were increased in carcinoma cells, while C6 regulatory T (Treg) cells, C11 T helper cell were decreased in carcinoma cells.

### C2 goblet cells are the key cell group in the process of carcinogenesis

DISEASE enrichment results showed that C2 cells significantly enriched in intestinal cancer, biliary tract cancer, appendix cancer, pseudomyxoma peritonei, bile duct adenocarcinoma, cholangiocarcinoma, bile duct cancer, bile duct carcinoma, colon cancer, et al. (Additional file 1: Fig.S1A). Hence, C2 goblet cells may be the main carcinogenic cell group. KEGG analysis showed that C2 group cells were significantly enriched in mucin-type O − glycan biosynthesis, *Vibrio cholerae* infection, complement and coagulation cascades, cardiac muscle contraction, hypertrophic cardiomyopathy (HCM). The main enriched functional genes are MUC2 [10], ST6GALNAC1 [11], SERPINA1 [12], and TPM1 [13] (Additional file 1: Fig.S1B). And the 6 coincident genes significantly enriched in adenoma and carcinoma were DEFA6 [14], SOX9 [15, 16], ID1 [17], L1TD1 [18], CXCL3 [19], and ODC1 [20] (Additional file 1: Fig.S1C, S1D). In addition, the similar analysis was performed in C3 cancer cells (Additional file 1: Fig.S2). The main enriched functional genes in C3 groups are SLC12A2 [21], ATP1B1 [22], PLA2G2A [23], and HBEGF [24]. (Additional file 1: Fig.S2B). And the 9 coincident genes significantly enriched in adenoma and carcinoma were APCDD1, DUOX2, NKD1, LYZ, CXCL1, TFF3, MSX1, CCL20, and IFI6 (Additional file 1: Fig.S2C, S2D).

### Gene expression and characteristics of epithelial cells during the progression of normal to adenoma and to carcinoma

**Fig. 3:**
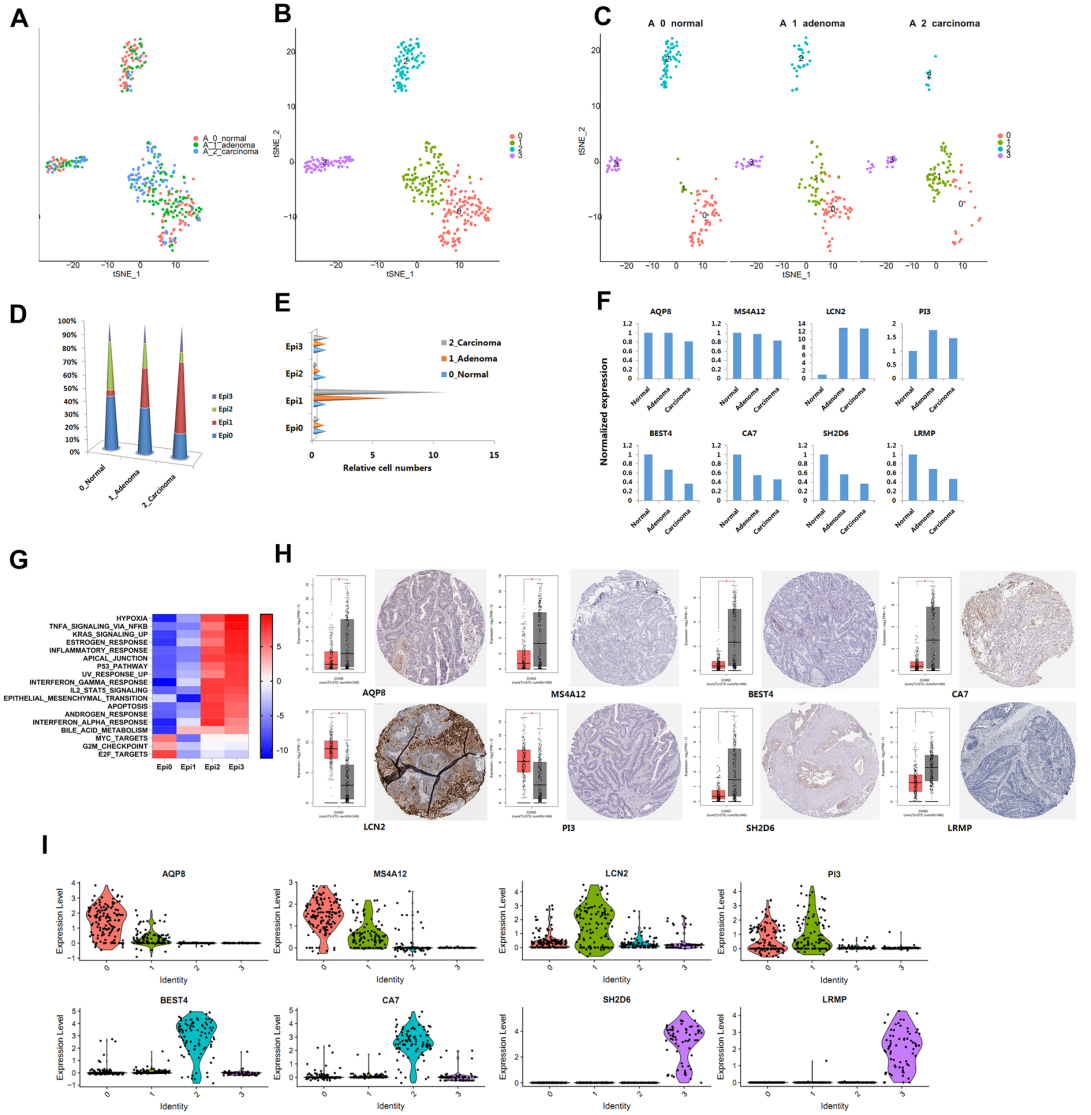
Identification of epithelial cell clusters and their expression features. **A**. tSNE plot of all the epithelial, colored by different stages. **B**. tSNE plot of all the epithelial, colored by clusters. **C**. tSNE plot of all the enterocyte, colored by different stages and clusters. **D**. The stacked histogram shows the 4 different cell cluster compositions in 3 pathological stages. **E**. The bar chart shows each enterocyte cell cluster composition of 3 pathological stages. **F**. The expression of marker genes of the enterocyte cells in different stages. **G**. Expression-based pathway activities scored by GSVA per enterocyte cluster, the red color indicates enrichment and blue color indicates not enrichment. **H**. The Top marker gene expression and cell location of epithelial clusters by TCGA and the Human Protein Atlas. **I** Violin plots of Top marker gene expression in enterocyte clusters

We identified 402 epithelial cells in all three stages (Fig. 3A), which were divided into four subtypes, named as Epi0, Epi1, Epi2, and Epi3 (Fig. 3B). Epithelial cells in three different pathological stages were analyzed in layers (Fig. 3C), and the number of cells in each layer was counted (Fig. 3D). In addition, we homogenized the proportion of cells between different groups compared with the normal group (Additional file 3: Table S2). Compared with normal tissues, the proportion of Epi1 cells in the adenoma tissues increased significantly and continued to increase in the carcinoma tissues, while the proportion of Epi3 cells was only increased slightly in carcinoma tissues. In addition, compared with normal tissues, the proportion of cells in Epi0 and Epi2 groups decreased in adenoma tissues and continued to decrease in carcinoma tissues (Fig. 3E). We made a histogram of the expression of top marker genes in different cell subsets at three pathological stages (Fig. 3F) and the heatmap of marker gene in all subsets (Additional file 1: Fig.S3A). We used the data of TCGA and HPA to verify the expression of these marker genes in colon cancer (Fig. 3H). The main marker genes are AQP8 [25], CA7 [26], MS4A12 [27], LRMP [28], LCN2 [29], and PI3 [30], BEST4, SH2D6 (no report is available about BEST4, SH2D6 and colon cancer).

We also conducted enrichment analysis of the four subsets of epithelial cells by GSVA method. Epi0 cells were enriched in myc targets, G2M check point, and E2F targets. Epi1 cells were enriched in bile acid metabolism. Epi2 and Epi3 cells showed similar expression patterns, Co-enriched in hypoxia, TNFa signaling via NFKB, KRAS signaling up, estrogen response, inflammatory response, apical junction, p53 pathway, UV response, interferon gamma response, IL2 stat5 signaling, epithelial mesenchymal transition, and bile acid metabolism (Fig. 3G). We also showed the expression of several marker genes in different epithelial cell subsets in the form of violin diagram (Fig. 3I).

As shown in Fig. 3D, E, the proportion of Epi1 cells increased significantly in the carcinoma tissue, suggesting that this group of cells may play a more important role in the evolution of tumor. The marker gene of the Epi1 subgroup is shown in Additional file 4: Table S3, Additional file 1: Fig.S3B. Some of them are involved in immuno reaction, such as IGKC [31], IGHA1, IGHA2, and JCHAIN [32]. In addition, evidence of involvement in carcinogenesis was found,some of them function as oncogenes in colon cancer including LCN2 [29], DUOX2 [33], CEACAM6 [34], CD55 [35], MUC5B [36], TM4SF1 [37], REG4 [38], TFF1 [39], GDF15 [40], and ANXA2 [41]. Some genes function as tumor suppressor including PLA2G2A [23, 42], PI3 [30], and NOS2 [43]. An association was found between TNFRSF6B SNP with Crohn’s disease susceptibility [44].

### Gene expression and characteristics of enterocyte cells during the progression of normal to adenoma and to carcinoma

**Fig. 4:**
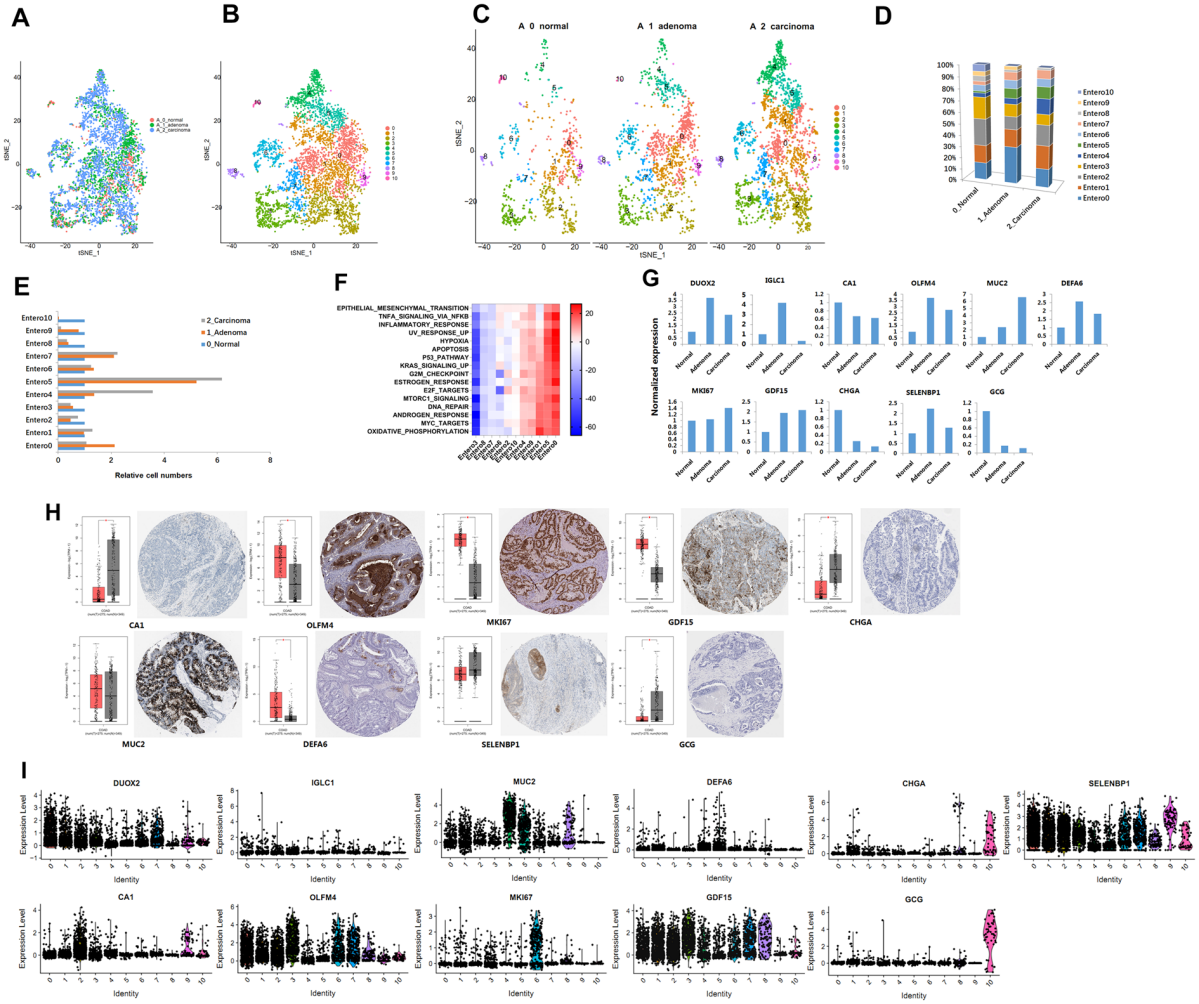
Identification of enterocyte cell clusters and their expression features. **A**. tSNE plot of all the enterocyte, colored by different stages. **B**. tSNE plot of all the enterocyte, colored by clusters. **C**. tSNE plot of all the enterocyte, colored by different stages and clusters. **D**. The stacked histogram shows the 11 different cell cluster composition in 3 pathological stages. **E**. The bar chart shows each enterocyte cell cluster composition of 3 pathological stages. **F**. The expression of marker genes of the enterocyte cells in different stages. **G**. Expression-based pathway activities scored by GSVA per enterocyte cluster, the red color indicates enrichment and blue color indicates not enrichment. **H**. The top marker gene expression and cell location of enterocyte clusters by TCGA and the Human Protein Atlas. **I**. Violin plots of top marker gene expression in enterocyte clusters

We identified 4835 enterocyte cells in all three stages (Fig. 4A). These enterocyte cells were divided into 11 subtypes named Entero0 to Entero10 (Fig. 4B). The enterocyte cells in three different pathological stages were analyzed in layers (Fig. 4C), and the number of cells in each layer was counted (Fig. 4D). In addition, we homogenized the proportion of cells in different groups in this sample (Additional file 3: Table S2, Fig. 4E). Compared with the normal tissue, the proportion of Entero4 and Entero5 groups in the adenoma tissue increased significantly and continued to increase significantly in the carcinoma tissue. The proportion of cells in Entero7 group increased significantly in adenoma and carcinoma tissues, while the proportion of cells was very close. Compared with the normal tissue, the proportion of Entero0 cells in the adenoma tissue increased significantly but decreased in the carcinoma tissue.

We made a histogram of the top gene expression of marker genes in different cell subsets at three pathological stages (Fig. 4G) and a heat map of marker genes in all subsets of cells (Additional file 1: Fig.S4A). We used the data of TCGA and HPA to verify the expression of these marker genes in colon cancer (Fig. 4H). The main marker genes are DUOX2 [33], OLFM4 [45], MUC2 [10], DEFA6 [14], MKI67, GDF15 [40], SELENBP1 [46], IGLC1 (no related study with colon cancer has been reported), CA1 [47], CHGA [48], and GCG [28].

Moreover, CA1, CHGA, and GCG decreased significantly from normal to adenoma and continued to decrease significantly from adenoma to carcinoma, indicating that these genes continued to play a role in inhibiting the process from normal to adenoma then to carcinoma. We also conducted enrichment analysis of 11 subsets of enterocyte cells by GSVA method. Cells in Entero0 and Entero5 groups had similar expression patterns, Co-enriched in EMT, TNFa signaling via NFKB, inflammatory response, UV response, hypoxia, apoptosis, p53 pathway, KRAS signaling, et al. and others. Entero1 was enriched in DNA repair, androgen response, MYC targets, and oxidative phosphorylation (Fig. 4F). We also showed the expression of several marker genes in different enterocyte cell subsets in the form of violin diagram (Fig. 4I).

As shown in Fig. 4E, the proportion of Entero0 and Entero5 cells increased significantly in the adenoma tissue, suggesting that these group of cells may play an important role in the evolution of tumor. The marker gene of Entero0 and Entero5 subgroups are shown in Additional file 4: Table S3, Additional file 1: Fig.S4B, S5A.

### Gene expression and characteristics of T cells during the progression of normal to adenoma and to carcinoma

**Fig. 5:**
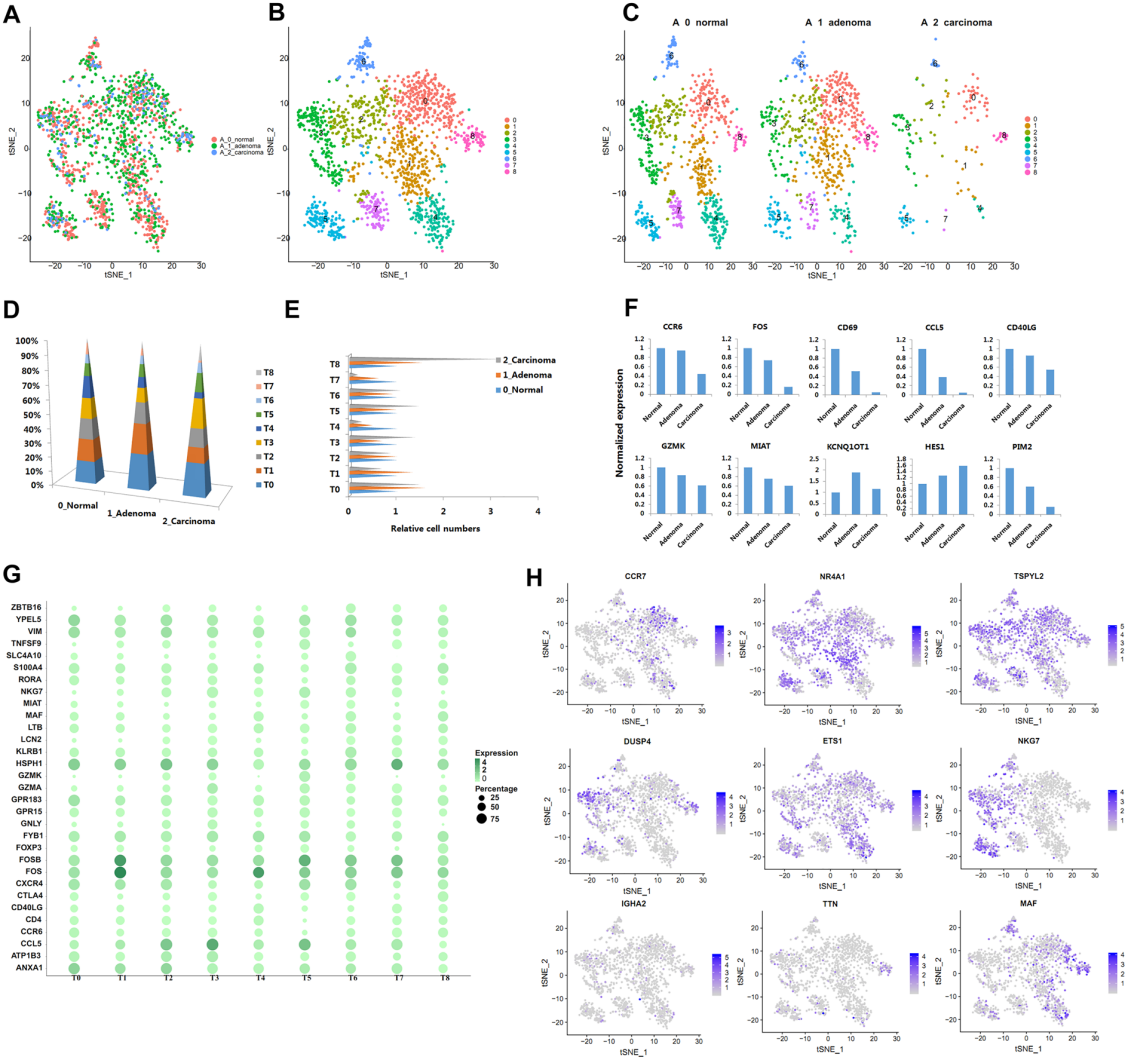
Identification of T cell clusters and their expression features. **A**. tSNE plot of all the T cell, colored by different stages. **B**. tSNE plot of all the T cell, colored by clusters. **C**. tSNE plot of all the T cell, colored by different stages and clusters. **D**. The stacked histogram shows the 9 different cell cluster composition in 3 pathological stages. **E**. The bar chart shows each T cell cluster compositions of 3 pathological stages. **F**. The expression of marker genes of the T cells in different stages. **G**. Bubble chart showed the top marker gene expression and percentage in different T cell clusters. **H**. tSNE plot showed the top marker gene expression in different T cell clusters

We identified 1555 T cells in all three stages (Fig. 5A). These T cells were divided into nine subtypes, named T0 to T8 (Fig. 5B). The T cells in three different pathological stages were analyzed in layers (Fig. 5C), and the marker gene heat map of all subpopulations of cells was prepared Additional file 1: Fig.S6A. The number of cells in each layer was counted (Fig. 5D). In addition, we homogenized the proportion of cells among different groups compared with the normal group (Additional file 3: Table S2, Fig. 5E). Compared with the normal group, the proportion of cells in T1, T2, T4, and T7, especially T4 and T7 groups, decreased significantly, suggesting that the number of cells in T4 and T7 groups decreased during carcinogenesis. Compared with the normal group, the proportion of cells inT0, T3, T5, and T8, especially T8 group, increased significantly, suggesting that the number of cells in the T8 group increased during carcinogenesis.

In Fig. 5F, we selected the top 2 gene in the marker gene of different subpopulations of cells, including CCR6, FOS, CD69, CCL5, CD40LG, GZMK, MIAT, PIM2, KCNQ1OT1, and HES1. Among them, CCR6, FOS, CD69, CCL5, CD40LG, GZMK, MIAT, PIM2 have lower expression in carcinoma tissues compared with normal tissues. However, KCNQ1OT1 and HES1 have higher expression in the carcinoma tissue compared with normal tissues. We selected the top 2 gene of each subgroup marker gene, prepared a bubble chart based on the positive expression cells and expression level (Fig. 5G), and selected the top gene of each subgroup marker gene by tSNE method for display (Fig. 5H).

In addition, based on the significant increase in the proportion of cells in the T8 group in cancer tissues, we further analyzed the marker gene of cells in the T8 group (Additional file 4: Table S3, Additional file 1: Fig.S6B). Among them, CTLA4 [49], TIGIT [50], and ICOS [51] are treated as immune checkpoint. GBP4 might be as potential novel immune checkpoint genes of CRC [52]. TTN [53], NAMPT [54], GBP4 [52], CTSC [55], and PIM2 [56] may be potential immunotherapy target. FOXP3 [57], CTLA4 [49], and PIM2 [56] mainly play inhibitory role to T cells. ICOS [51], MAF [58] mainly activate or preserve the function of Treg cells. Moreover, the function of TBC1D4, LTB, C2CD4A, GBP4/5 in T cells needs to be clarified further.

### Cell fate differentiation based on enterocyte cells

**Fig. 6:**
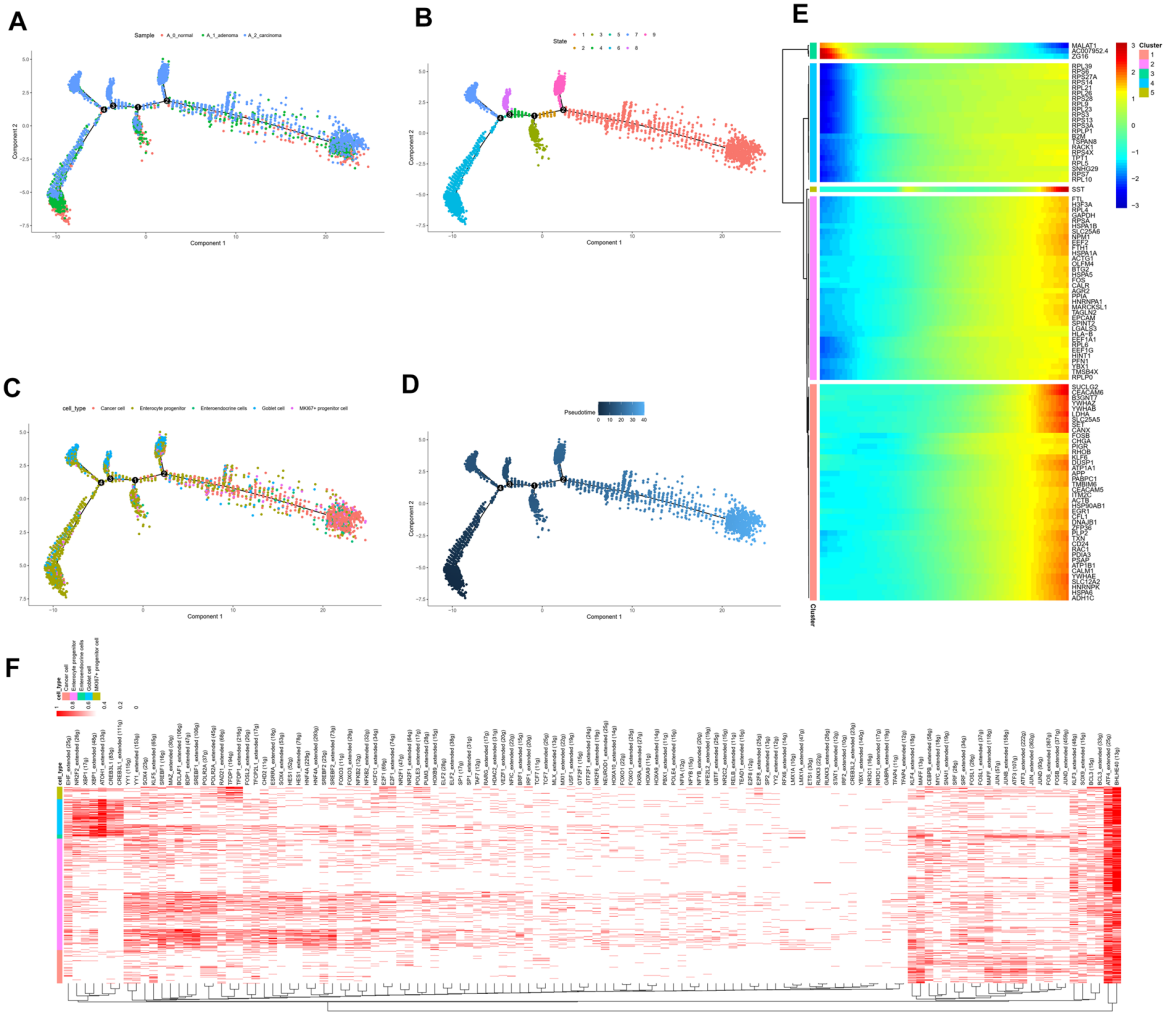
Cell fate differentiation based on enterocyte cells. **A**. Trajectories of enterocyte cells differentiated by samples. **B**. Trajectories of the enterocyte cells differentiated by states. **C**. Trajectories of the enterocyte cells differentiated by clusters. **D**. Pseudo temporal trajectory analysis of enterocyte cells. **E**. The top100 different expressed gene heatmap by pseudotime. **F**. Transcription factors regulated the cancer-related genes

Basing on the trajectory plots of enterocyte cells differentiated by sample, we can observe bifurcation (Fig. 6A). Pseudotime trajectory analysis reveals 9 states of enterocyte cells (Fig. 6B). 5 distinct cell clusters are present in enterocyte cells. Trajectory plots differentiated by cluster reveal the distribution of each cluster in pseudotime trajectory (Fig. 6C), and data showed that colon cancer cells were derived from enterocyte progenitor cells. By pseudo temporal trajectory analysis of the single-cell transcriptomes, we can observe the evolution of cells. (Fig. 6D). The top100 differentially expressed gene heatmap by pseudotime which might be associated with cancer lineage is shown (Fig. 6E). We found lots of them might play a key role in the transformation of enterocyte progenitor cells to cancer cells such as MALAT1 [59], B2M [60], EEF1A1 [61], RPL5 [62], B3GNT7 [63], RHOB [64] and ACTB [65] might play a key role in the transformation of enterocyte progenitor cells to cancer cells. Transcription factor regulated these cancer-related genes were presented in Fig. 6F. In addition, cell fate differentiation based on all the cells are shown in Additional file 1: Fig.S7.

### The expression and clinical significance of the new discovered genes in colon cancer

**Fig.7:**
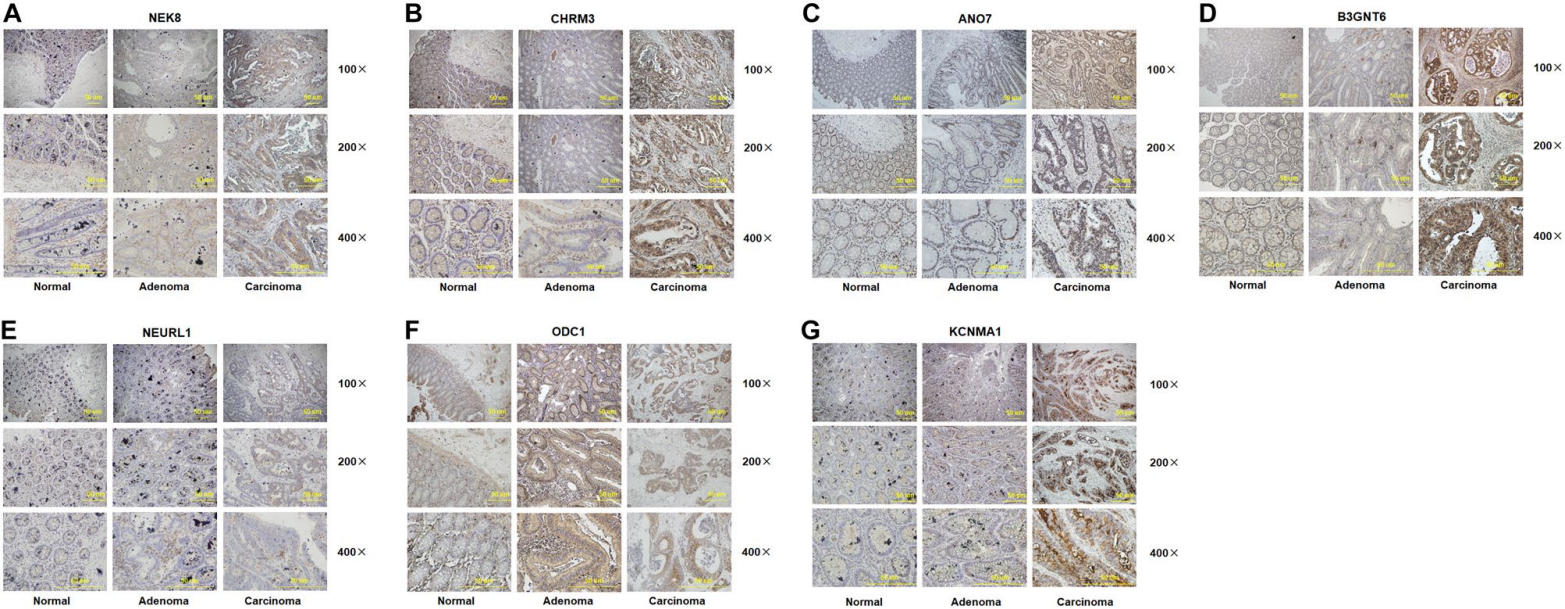
The expression and clinical significance of the new discovered genes in colon cancer. **A**–**G**. Immunohistochemical staining was performed of 7 selected genes including NEK8, CHRM3, ANO7, B3GNT6, NEURL1, ODC1 and KCNMA1 in colon normal tissue, adenoma tissue and carcinoma tissues

We collected the resected carcinoma tissues from CRC patients and conducted immunohistochemical (IHC) staining analysis to examine the expression of some representative genes including NEK8, CHRM3, ANO7, B3GNT6, NEURL1, ODC1 and KCNMA1 in colon normal tissue, adenoma tissue and carcinoma tissues. The immunohistochemistry images below showed that all of these proteins were highly expressed in carcinoma tissues compared with the corresponding normal and adenoma tissues (Fig. 7A–G).

## Discussion

In this study, we used high-throughput scRNA-seq analysis and drew a complete atlas of transitional fate cells, characterized the transcriptome characteristics of cell clusters from normal colon to adenoma, then to carcinoma, and clarified the possible evolution trajectory of cells in the multi-step process. In the evolution process of colon cancer, we mainly focused on the expression transformation of epithelial cells, intestinal cells, and T cells related to tumorigenesis since how they change from normal colon to adenoma to carcinoma phenotype is a long-standing problem.

The constituent ratio of C2 goblet cells was significantly higher in cancer tissues than in normal tissues, suggesting that it may be a key group of carcinogenic cells. DISEASE enrichment results clearly showed that these cells were significantly enriched in a variety of tumors, including gastrointestinal tumors. KEGG analysis showed that C2 goblet cells were specially significantly enriched in the function of mucin type O-glycan biosynthesis (Additional file 1: Fig.S1B). MUC2 [10], ST6GALNAC1 [11], SERPINA1 [12], and TPM1 [13] play key roles in C2 goblet cells. In C2 goblet cells, during the process from normal colon to adenoma and from adenoma to carcinoma, several continuously highly expressed genes were found, including DEFA6 [14], SOX9 [15, 16], ID1 [17], L1TD1 [18], CXCL3 [19], and ODC1 [20]. No report is available about ODC1, and colon cancer. Further studies should explore the association among ODC1, and colon cancer.

By analyzing the subsets of epithelial cell, the cells in Epi1 group highly increased in cancer tissue, suggesting that Epi1 group cells may play a key carcinogenic role in epithelial cell population. GO enrichment analysis of marker gene of Epi1 group cells found that, IGKC, LCN2, PI3, JCHAIN, IGHA1, PLA2G2A, CD55, and IGHA2 are related to humoral immune response. LCN2, PI3, JCHAIN, IGHA1, PLA2G2A, and IGHA2 are related to antimicrobial humoral response. IGKC, LCN2, PI3, JCHAIN, IGHA1, PLA2G2A, IGHA2, and NOS2 are related to defense response to bacterium. IGKC, HBA2, HBA1, JCHAIN, IGHA1, IGHA2, and ANXA2 are related to receptor-mediated endocytosis (Additional file 1: Fig.S3C). KEGG analysis showed that LCN2, MUC5B are related to IL-17 signaling pathway (Additional file 1: Fig.S3D). These enrichments suggesting that antimicrobial and IL-17 signaling pathway are very important in Epi1 cells, and IGKC, LCN2, PI3, JCHAIN, IGHA1, PLA2G2A, CD55, IGHA2, NOS2, ANXA2, and MUC5B are the key oncogenes in Epi1 cells.

By analyzing the subsets of enterocyte cells, we found that most of these subsets are involved in the process of carcinogenesis. We think Entero0 and Entero5 subsets were the more important cell groups of enterocyte cells, since the cell percentages were significantly increased in Entero0 and Entero5 in carcinoma tissues compare to normal colon. Observing the top 20 marker genes of Entero0, GO analysis showed that LCN2, PI3, NOS2, PLA2G2A, CCL20 are related with defense response to bacterium. LCN2, PI3, CXCL1, PLA2G2A are related with humoral immune response and antimicrobial humoral response (Additional file 1: Fig.S4C). KEGG analysis showed that these genes enriched in IL-17 signaling pathway, pancreatic secretion, and amoebiasis (Additional file 1: Fig.S4D). These findings suggested that antimicrobial and IL-17 signaling pathway are the most important signaling in Entero0, and LCN2, PI3, NOS2, PLA2G2A, CCL20, and CXCL1 were the key oncogenes. Observing the Top 20 marker genes of Entero5, the GO analysis showed that DEFA6, LCN2, WFDC2, and SPNS2 are related to humoral immune response (Additional file 1: Fig.S5B). KEGG analysis showed that genes including ST6GALNAC1 and B3GNT6 are enriched in mucin type O-glycan biosynthesis, CLCA1 and KCNMA1 enriched in renin secretion and pancreatic secretion (Additional file 1: Fig.S5C). These findings suggested that immune response and mucin type O-glycan biosynthesis are the very important signaling in Entero5. And DEFA6, LCN2, WFDC2, SPNS2, ST6GALNAC1, B3GNT6, CLCA1, and KCNMA1 may play key roles in colon carcinogenesis. Moreover, the association between KCNMA1, B3GNT6, and colon cancer needs to be explored further. we performed copy number variation (CNV) analysis and identified cancer cells harboring chromosomal abnormalities. These cancer cells were grouped together with the enterocyte progenitor, goblet, and enteroendocrine cells at low resolution (Fig. 1D).

As a whole, the number of T cell subsets decreased significantly from normal to adenoma to carcinoma, suggesting that the decline of T cell function is an important factor in the development of tumor. While observing the cell number composition ratio of T cell subsets, we found the composition ratio of cell number were varied from normal colon to adenoma to carcinoma. Compared with the cell proportion in normal colon, T1, T2, T4, and T7 cell sets were significantly decreased in carcinoma tissues, especially T4 and T7, while the cell proportion of T0, T3, T5 and T8 cell sets increased significantly, especially T8. Observing the marker gene of T8 group cells (Additional file 4: Table S3, Additional file 1: Fig.S6B), GO enrichment analysis showed that FOXP3, CTLA4, TIGIT, TNFRSF1B, and ICOS are enriched in T cell activation and regulation of T cell activation, FOXP3, BIRC3, CTSC, TNFRSF1B, and C2CD4A are enriched in regulation of inflammatory response (Additional file 1: Fig.S6C). KEGG analysis showed that BIRC3, NAMPT, GBP4, and GBP5 are enriched in NOD-like receptor signaling pathway. LTB, TNFRSF1B, CXCR6, and CCL20 are enriched in cytokine–cytokine receptor interaction (Additional file 1: Fig.S6D).

In conclusion, we performed single-cell transcriptomic analysis on the same patient who carried normal colon tissue, adenoma tissue, and carcinoma tissue at the same time. Due to the same microenvironment, we perfectly revealed the evolution process of time-dependent colon cancer. By analyzing various cell types at different pathological stages, we drew the detailed evolution map, and some key expression characteristics were identified. Our results confirmed that there were dominant clones of similar cells in the process of carcinogenesis, and the dominant cloned cells in this study were C2 goblet cell group. In addition, there were also dominant clones of similar cells in epithelial cells and enterocyte cells, Epi1, Entero0, and Entero5 groups. After comprehensive analysis of the enriched genes of the dominant cloned cells in this study, we found that mucin type O-glycan biosynthesis, antibacterial, and IL-17 signaling pathway are important pathways of colon carcinogenesis, especially mucin type O-glycan biosynthesis, which deserves attention in further study. Mucins are the main components of mucus, which is secreted by goblet cells and forms a protective homeostatic barrier between the resident microbiota and the underlying immune cells in the colon, mucin type O-glycan and goblet cells have made great contributions to maintaining the homeostasis of colonic mucosa [66, 67]. It is suggested that if we focus on the function of goblet cells and mucin-type O-glycanbiosynthesis, there may be important discoveries in the prevention and treatment of colon cancer. At the same time, some genes including AC007952.4, NEK8, CHRM3, ANO7, B3GNT6, NEURL1, ODC1, and KCNMA1 may play critical role in colon carcinogenesis, which need to be studied further.

## Supplementary Information

**Additional file 1: Figure.S1**. Enrichments analysis in cluster 2 goblet cells. A. Disease enrichment of the genes in cluster 2 goblet cells. B. KEGG enrichment of the genes in cluster 2. C. The Top 20 genes in adenoma tissue compare to normal tissue in cluster 2. D. The Top 20 genes in adenoma tissue compare to normal tissue in cluster 2. **Figure.S2**. Enrichments analysis in cluster 3 colon cancer cells. A. Disease enrichment of the genes in cluster 3 colon cancer cells. B. KEGG enrichment of the genes in cluster 3. C. The top 20 genes in adenoma tissue compare to normal tissue in cluster 3. D. The Top 20 genes in adenoma tissue compare to normal tissue in cluster 3. **Figure S3**. characteristics of epithelial cell subsets and Epi1. A. The maker genes of the four epithelial cell subsets. B. The top marker genes of the Epi1 subset. C. The most enriched signaling of Epi1 by GO enrichment analysis. D. The most enriched signaling of Epi1 by KEGG enrichment analysis. **Figure.S4**. characteristics of enterocyte cell subsets and Entero0. A. The maker genes of the eleven epithelial cell subsets. B. The top marker genes of the Entero0 subset. C. The most enriched signaling of Entero0 by GO enrichment analysis. D. The most enriched signaling of Entero0 by KEGG enrichment analysis. **Figure.S5**. characteristics of enterocyte cell subsets and Entero5. A The top marker genes of the Entero5 subset. B. The most enriched signaling of Entero5 by GO enrichment analysis. C. The most enriched signaling of Entero5 by KEGG enrichment analysis. **Figure.S6**. characteristics of T cell subsets and T8. A The maker genes of the nine T cell subsets. B. The top marker genes of the T8 subset. C. The most enriched signaling of T8 by GO enrichment analysis. D. The most enriched signaling of T8 by KEGG enrichment analysis. **Figure.S7**. Cell fate differentiation based on all the cells. A. Single cell trajectories differentiated by samples. B. Single cell trajectories differentiated by states. C. Single cell trajectories differentiated by clusters. D. Cell pseudo temporal trajectory analysis. E. Trajectory graph showed by each sample. F. The top50 different expressed gene heatmap by pseudotime. G. The top50 different expressed gene heatmap by branch.

**Additional file 2: Table S1. **The marker genes of the different cell types.

**Additional file 3: Table S2. **The cell number and proportion of all types of cells.

**Additional file 4: Table S3. **The Top20 genes of cell subsets.
