# Supplementary material for: Single-cell transcriptomic analysis of normal and pathological tissues from the same patient uncovers colon cancer progression
Source: Cell Biosci. 2023 Mar 21;13:62. doi: 10.1186/s13578-023-01002-w (PMC10031920; doi:10.1186/s13578-023-01002-w)
Supplement: Supplementary file 1 — Additional file 1: Figure.S1. Enrichments analysis in cluster 2 goblet cells. A. Disease enrichment of the genes in cluster 2 goblet cells. B. KEGG enrichment of the genes in cluster 2. C. The Top 20 genes in adenoma tissue compare to normal tissue in cluster 2. D. The Top 20 genes in adenoma tissue compare to normal tissue in cluster 2. Figure.S2. Enrichments analysis in cluster 3 colon cancer cells. A. Disease enrichment of the genes in cluster 3 colon cancer cells. B. KEGG enrichment of the genes in cluster 3. C. The top 20 genes in adenoma tissue compare to normal tissue in cluster 3. D. The Top 20 genes in adenoma tissue compare to normal tissue in cluster 3. Figure S3. characteristics of epithelial cell subsets and Epi1. A. The maker genes of the four epithelial cell subsets. B. The top marker genes of the Epi1 subset. C. The most enriched signaling of Epi1 by GO enrichment analysis. D. The most enriched signaling of Epi1 by KEGG enrichment analysis. Figure.S4. characteristics of enterocyte cell subsets and Entero0. A. The maker genes of the eleven epithelial cell subsets. B. The top marker genes of the Entero0 subset. C. The most enriched signaling of Entero0 by GO enrichment analysis. D. The most enriched signaling of Entero0 by KEGG enrichment analysis. Figure.S5. characteristics of enterocyte cell subsets and Entero5. A The top marker genes of the Entero5 subset. B. The most enriched signaling of Entero5 by GO enrichment analysis. C. The most enriched signaling of Entero5 by KEGG enrichment analysis. Figure.S6. characteristics of T cell subsets and T8. A The maker genes of the nine T cell subsets. B. The top marker genes of the T8 subset. C. The most enriched signaling of T8 by GO enrichment analysis. D. The most enriched signaling of T8 by KEGG enrichment analysis. Figure.S7. Cell fate differentiation based on all the cells. A. Single cell trajectories differentiated by samples. B. Single cell trajectories differentiated by states. C. Single cell trajecto [file 13578_2023_1002_MOESM1_ESM.docx]

**Supplementary Figure legend**

**Supplementary Fig.S1. Enrichments analysis in cluster 2 goblet cells.**

A. Disease enrichment of the genes in cluster 2 goblet cells. B. KEGG enrichment of the genes in cluster 2. C. The Top 20 genes in adenoma tissue compare to normal tissue in cluster 2. D. The Top 20 genes in adenoma tissue compare to normal tissue in cluster 2.

**
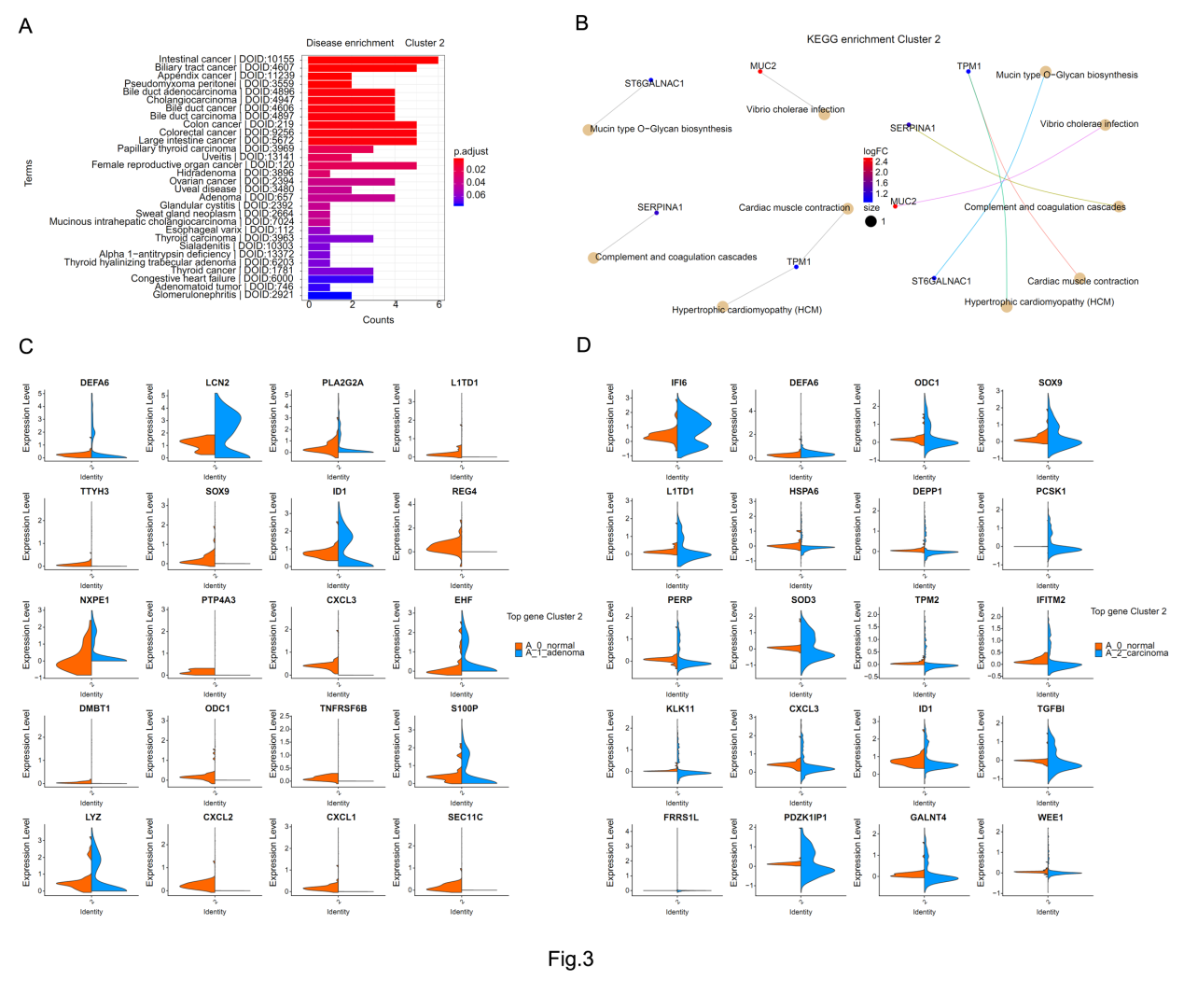
**

**Supplementary Fig.S2. Enrichments analysis in cluster 3 colon cancer cells.**

**A.** Disease enrichment of the genes in cluster 3 colon cancer cells. B. KEGG enrichment of the genes in cluster 3. C. The top 20 genes in adenoma tissue compare to normal tissue in cluster 3. D. The Top 20 genes in adenoma tissue compare to normal tissue in cluster 3.

**
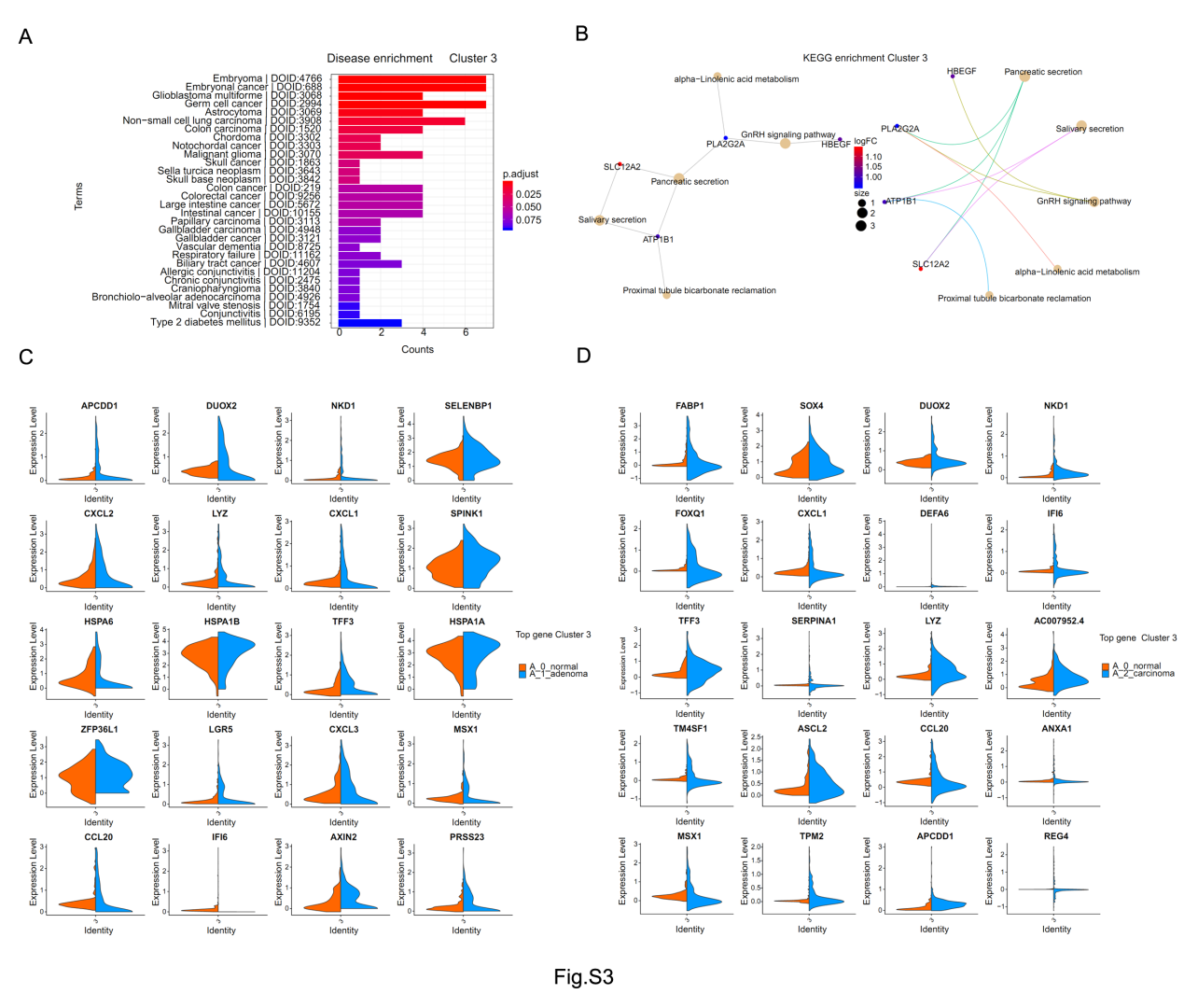
**

**Supplementary Figure S3. characteristics of epithelial cell subsets and Epi1.**

A. The maker genes of the four epithelial cell subsets. B. The top marker genes of the Epi1 subset. C. The most enriched signaling of Epi1 by GO enrichment analysis. D. The most enriched signaling of Epi1 by KEGG enrichment analysis.

**
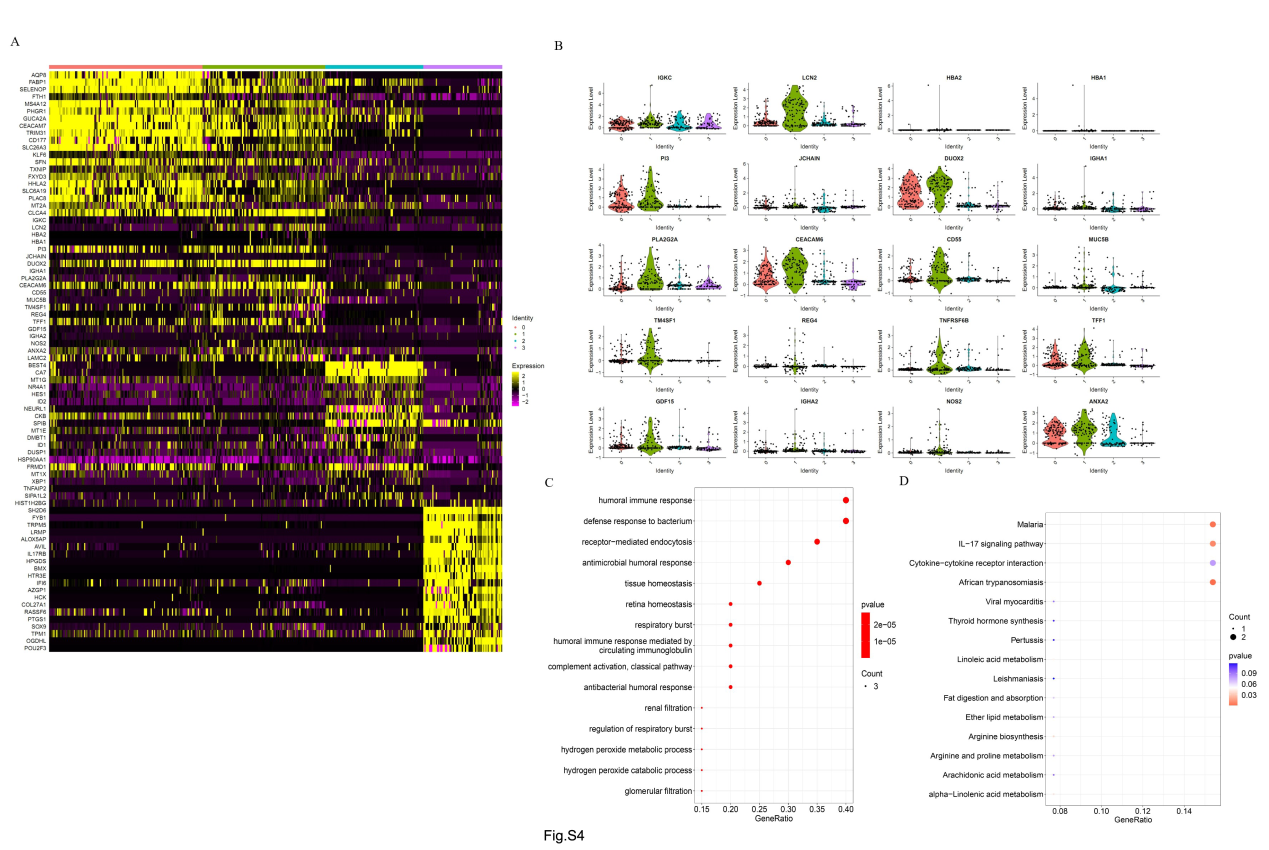
**

**Supplementary Fig.S4. characteristics of enterocyte cell subsets and Entero0.**

1. The maker genes of the eleven epithelial cell subsets. B. The top marker genes of the Entero0 subset. C. The most enriched signaling of Entero0 by GO enrichment analysis. D. The most enriched signaling of Entero0 by KEGG enrichment analysis.

**
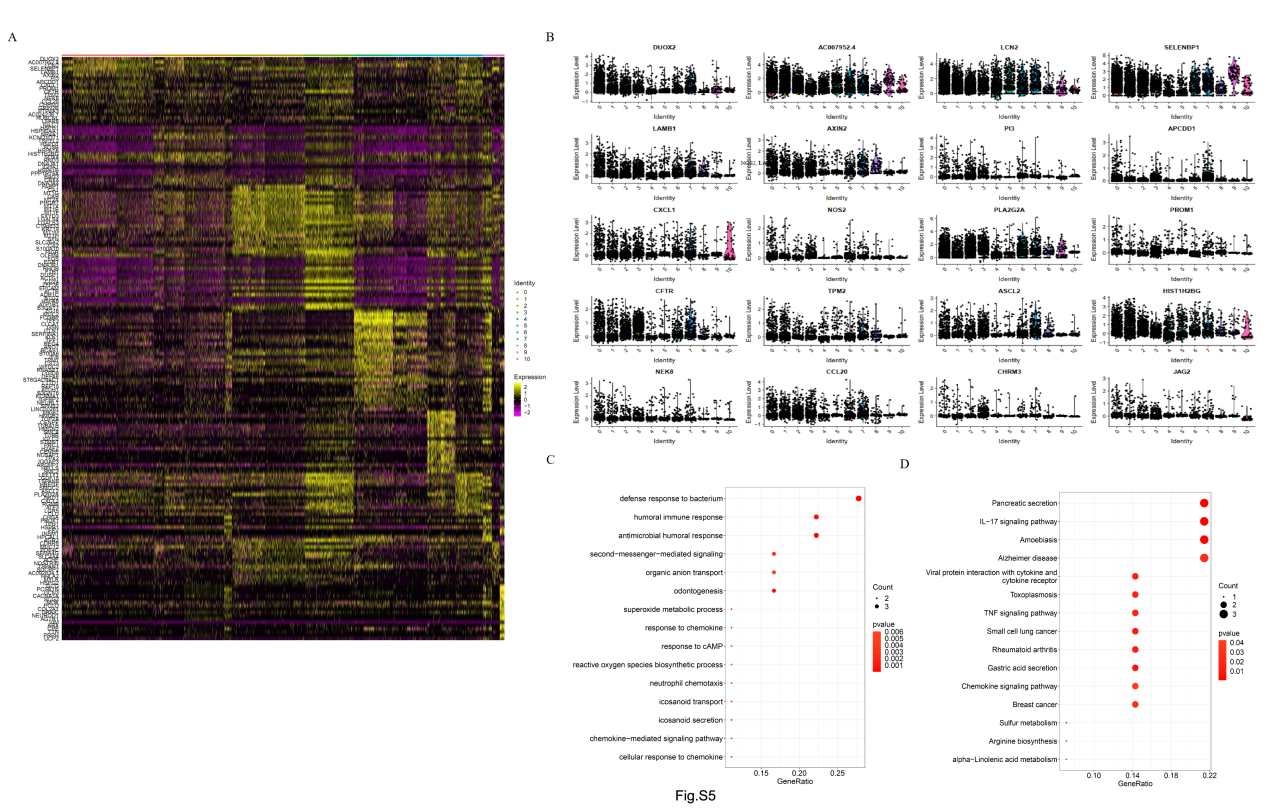
**

**Supplementary Fig.S5. characteristics of enterocyte cell subsets and Entero5.**

1. The top marker genes of the Entero5 subset. B. The most enriched signaling of Entero5 by GO enrichment analysis. C. The most enriched signaling of Entero5 by KEGG enrichment analysis.

**
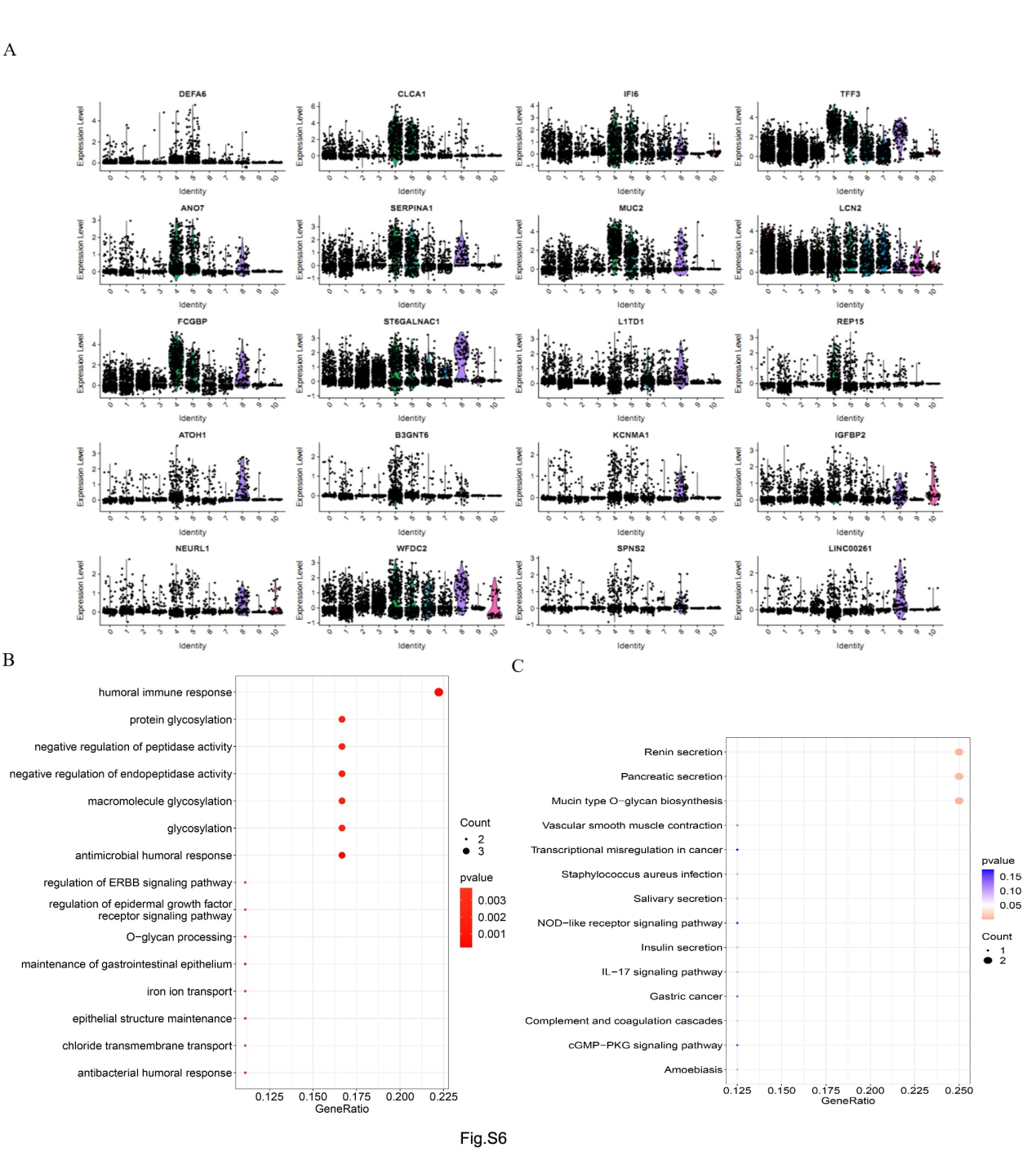
**

**Supplementary Fig.S6. characteristics of T cell subsets and T8.**

1. The maker genes of the nine T cell subsets. B. The top marker genes of the T8 subset. C. The most enriched signaling of T8 by GO enrichment analysis. D. The most enriched signaling of T8 by KEGG enrichment analysis.

**
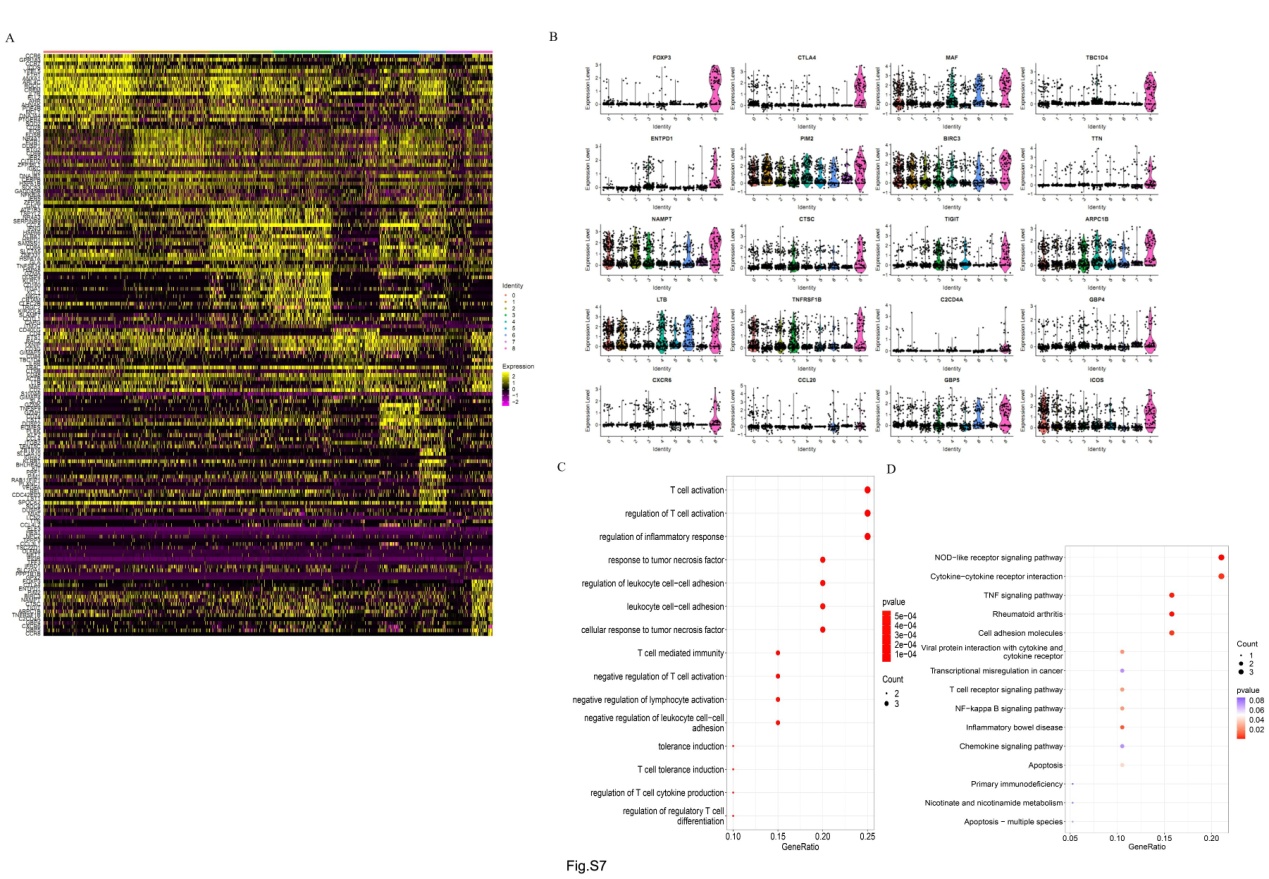
**

**Supplementary Fig.S7. Cell fate differentiation based on all the cells**

A. Single cell trajectories differentiated by samples. B. Single cell trajectories differentiated by states. C. Single cell trajectories differentiated by clusters. D. Cell pseudo temporal trajectory analysis. E. Trajectory graph showed by each sample. F. The top50 different expressed gene heatmap by pseudotime. G. The top50 different expressed gene heatmap by branch.
