# Supplementary material for: Single-cell transcriptomic analysis of normal and pathological tissues from the same patient uncovers colon cancer progression
Source: Cell Biosci. 2023 Mar 21;13:62. doi: 10.1186/s13578-023-01002-w (PMC10031920; doi:10.1186/s13578-023-01002-w)
Supplement: Supplementary file 2 — Additional file 2: Table S1. The marker genes of the different cell types. [file 13578_2023_1002_MOESM2_ESM.docx]

**Supplementary Table S1. The marker genes of the different cell types**

| **Cluster no.** | **Cell types** | **Marker genes** | | | | | | | | | |
| --- | --- | --- | --- | --- | --- | --- | --- | --- | --- | --- | --- |
| C0 | Enterocyte progenitor | LCN2 | PLA2G2A |  |  |  |  |  |  |  |  |
| C1 | Enterocyte progenitor | PLA2G2A |  |  |  |  |  |  |  |  |  |
| C2 | Goblet cell | ZG16 | CLCA1 | MUC2 | FCGBP | GSN | ANO7 | KLK1 | TFF3 |  |  |
| C3 | Cancer stem cell | OLFM4 | GDF15 | TSPAN8 | CD24 | EPCAM |  |  |  |  |  |
| C4 | Effector memory T cell | IL7R |  |  |  |  |  |  |  |  |  |
| C5 | Plasma cell | IGKC | JCHAIN | IGHA2 | IGHM | IGHG4 | IGHA1 | IGKV4-1 | TXNDC5 | IGHG2 | IGHG1 |
| C6 | Regulatory T (Treg) cell | TRBC2 | DUSP4 | TSPYL2 |  |  |  |  |  |  |  |
| C7 | MKI67+ progenitor cell | MKI67 | TOP2A | CENPF | PRC1 |  |  |  |  |  |  |
| C8 | Mast cell | TPSB2 | TPSAB1 | CPA3 | MS4A2 | CSF1 | CLU | KIT | CTSG | GATA2 |  |
| C9 | Absorptive cell | DUOX2 | AQP8 | MS4A12 | PI3 | SLC26A3 | KRT20 |  |  |  |  |
| C10 | B cell | IGLC2 | IGLC3 | IGLV2-14 | IGLV6-57 | JCHAIN | IGHA2 |  |  |  |  |
| C11 | T helper cell | TRBC2 | TRAC | MAF |  |  |  |  |  |  |  |
| C12 | Dentritic cell | HLA-DRA | HLA-DRB1 | HLA-DRB5 | CD83 |  |  |  |  |  |  |
| C13 | Macrophage | CTSB | CCL4L2 | CCL4 | CTSD | LYZ |  |  |  |  |  |
| C14 | CD8+ T cell | CCL5 | GZMK | NKG7 | TRBC2 | CD8A |  |  |  |  |  |
| C15 | Enteroendocrine cells | CHGA |  |  |  |  |  |  |  |  |  |
| C16 | Natural killer T (NKT) cell | KLRB1 | SPOCK2 | TNFAIP3 | RUNX3 | ZBTB16 | CXCR4 | ZNF331 | PIM1 |  |  |
| C17 | Epithelial cell | CA7 | GUCA2B | SPIB | CA1 | KRT20 |  |  |  |  |  |
| C18 | Epithelial cell | SOX9 | AOC1 | SPIB | HEPACAM2 |  |  |  |  |  |  |
| C19 | Granulocytes | CXCL8 | S100A9 | IFITM2 | NAMPT | C5AR1 | FCGR3B | PTGS2 | IL1B | S100A8 | G0S2 |
| C20 | Fibrocyte | COL3A1 | COL1A1 | SPARCL1 | DCN | MMP2 |  |  |  |  |  |
| C21 | Enterocyte progenitor | CHGA | NEUROD1 | CPE |  |  |  |  |  |  |  |
