# Supplementary material for: Single-cell transcriptomic analysis of normal and pathological tissues from the same patient uncovers colon cancer progression
Source: Cell Biosci. 2023 Mar 21;13:62. doi: 10.1186/s13578-023-01002-w (PMC10031920; doi:10.1186/s13578-023-01002-w)
Supplement: Supplementary file 3 — Additional file 3: Table S2. The cell number and proportion of all types of cells. [file 13578_2023_1002_MOESM3_ESM.docx]

**Supplementary Table S2. The cell number and proportion of all types of cells**

|  |  |  | **Cell No.(%)** | | |
| --- | --- | --- | --- | --- | --- |
|  | **Cluster** | **Cell No.** | **0_normal** | **1_polyp** | **2_carcinoma** |
| **All the cells** | **C0** | 1820 | 253 (11.0) | 715 (25.2) | 852 (28.3) |
|  | **C1** | 870 | 223 (9.7) | 199 (7.0) | 448 (14.9) |
|  | **C2** | 867 | 51 (2.2) | 197 (6.9) | 619 (20.6) |
|  | **C3** | 822 | 180 (7.8) | 248 (8.7) | 394 (13.1) |
|  | **C4** | 692 | 288 (12.5) | 341 (12.0) | 63 (2.1) |
|  | **C5** | 410 | 168 (7.3) | 173 (6.1) | 69 (2.3) |
|  | **C6** | 389 | 229 (9.9) | 121 (4.3) | 39 (1.3) |
|  | **C7** | 306 | 43 (1.9) | 103 (3.6) | 160 (5.3) |
|  | **C8** | 265 | 89 (3.9) | 153 (5.4) | 23 (0.8) |
|  | **C9** | 245 | 69 (3.0) | 95 (3.4) | 81 (2.7) |
|  | **C10** | 238 | 98 (4.2) | 93 (3.3) | 47 (1.6) |
|  | **C11** | 238 | 140 (6.1) | 76 (2.7) | 22 (0.7) |
|  | **C12** | 165 | 121 (5.2) | 26 (0.9) | 18 (0.6) |
|  | **C13** | 160 | 49 (2.1) | 67 (2.4) | 44 (1.5) |
|  | **C14** | 143 | 69 (3.0) | 56 (2.0) | 18 (0.6) |
|  | **C15** | 99 | 37 (1.6) | 26 (0.9) | 36 (1.2) |
|  | **C16** | 93 | 47 (2.0) | 35 (1.2) | 11 (0.4) |
|  | **C17** | 87 | 53 (2.3) | 26 (0.9) | 8 (0.3) |
|  | **C18** | 70 | 24 (1.0) | 23 (0.8) | 23 (0.8) |
|  | **C19** | 59 | 8 (0.3) | 38 (1.3) | 13 (0.4) |
|  | **C20** | 59 | 19 (0.8) | 21 (0.7) | 19 (0.6) |
|  | **C21** | 51 | 48 (2.1) | 3 (0.1) | 0 |
| **Epithelial cells** | **Epi0** | 136 | 62 (42.5) | 52 (36.1) | 22 (19.6) |
|  | **Epi1** | 109 | 7 (4.79) | 43 (29.8) | 59 (52.7) |
|  | **Epi2** | 87 | 53 (36.3) | 26 (18.1) | 8 (7.1) |
|  | **Epi3** | 70 | 24 (16.4) | 23 (16.0) | 23 (20.5) |
| **Enterocyte progenitor cells** | **Entero0** | 1000 | 125 (15.0) | 476 (31.9) | 399 (15.9) |
|  | **Entero1** | 860 | 130(15.6) | 227 (15.2) | 503 (20.0) |
|  | **Entero2** | 799 | 195 (23.3) | 164 (11.0) | 440 (17.5) |
|  | **Entero3** | 534 | 156 (18.7) | 156 (10.4) | 222 (8.8) |
|  | **Entero4** | 425 | 30 (3.6) | 73 (4.9) | 322 (12.8) |
|  | **Entero5** | 375 | 13 (1.6) | 121 (8.1) | 241 (9.6) |
|  | **Entero6** | 306 | 43 (5.1) | 103 (6.9) | 160 (6.4) |
|  | **Entero7** | 299 | 26 (3.1) | 98 (6.6) | 175 (7.0) |
|  | **Entero8** | 100 | 37 (4.4) | 26 (1.7) | 37 (1.5) |
|  | **Entero9** | 86 | 32 (3.8) | 44 (3.0) | 10 (0.4) |
|  | **Entero10** | 51 | 48 (5.7) | 3 (0.2) | 0 |
| **T cells** | **T0** | 309 | 119 (15.4) | 155 (24.6) | 35 (22.9) |
|  | **T1** | 266 | 120 (15.5) | 130 (20.7) | 16 (10.5) |
|  | **T2** | 220 | 111 (14.4) | 90 (14.3) | 19 (12.4) |
|  | **T3** | 201 | 110 (14.2) | 61 (9.7) | 30 (19.6) |
|  | **T4** | 167 | 116 (15) | 45 (7.2) | 6 (3.9) |
|  | **T5** | 138 | 66 (8.5) | 53 (8.4) | 19 (12.4) |
|  | **T6** | 92 | 47 (6.1) | 35 (5.6) | 10 (6.5) |
|  | **T7** | 90 | 59 (7.6) | 29 (4.6) | 2 (1.3) |
|  | **T8** | 72 | 25 (3.2) | 31 (4.9) | 16 (10.5) |
