# Supplementary material for: Single-cell transcriptomic analysis of normal and pathological tissues from the same patient uncovers colon cancer progression
Source: Cell Biosci. 2023 Mar 21;13:62. doi: 10.1186/s13578-023-01002-w (PMC10031920; doi:10.1186/s13578-023-01002-w)
Supplement: Supplementary file 4 — Additional file 4: Table S3. The Top20 genes of cell subsets. [file 13578_2023_1002_MOESM4_ESM.docx]

**Supplementary Table S3. The Top20 genes of cell subsets**

|  | **Gene** | **Function** | **Role in colon cancer** |
| --- | --- | --- | --- |
| **Epithelial cell subset 1 (Epi1)** | IGKC (25) | Diseases associated with IGKC include Immunoglobulin Kappa Light Chain Deficiency and Plasma Cell Tumor |  |
|  | LCN2 (23) | An innate immune protein, might serve as a biomarker for immune infiltration and poor prognosis in cancers | Oncogene |
|  | HBA2 | Members of hemoglobin, diseases associated with HBA2 include Hemoglobin H Disease and Alpha-Thalassemia | Unknown |
|  | HBA1 | Members of hemoglobin, diseases associated with HBA1 include Erythrocytosis, Familial, 7 and Hemoglobin H Disease. | Unknown |
|  | PI3 (24) | Functions as an antimicrobial peptide against Gram-positive and Gram-negative bacteria, and fungal pathogens |  |
|  | JCHAIN (26) | Diseases associated with JCHAIN include Alpha Chain Disease and Climatic Droplet Keratopathy |  |
|  | DUOX2 (27) | A member of the NADPH oxidase family, promotes the progression of colorectal cancer cells by regulating the AKT pathway and interacting with RPL3 | Oncogene |
|  | IGHA1 | Among its related pathways are Regulation of nuclear SMAD2/3 signaling and Binding and Uptake of Ligands by Scavenger Receptors |  |
|  | PLA2G2A (16, 36) | Secretory calcium-dependent phospholipase A2, could reduce colon tumorigenesis independent of Apc-germline mutations | Tumor suppressor |
|  | CEACAM6 (28) | Belongs to the carcinoembryonic antigen (CEA) family, play a role in cell adhesion and are widely used as tumor markers in serum immunoassay determinations of carcinoma | Oncogene |
|  | CD55 (29) | Involved in the regulation of the complement cascade | Oncogene |
|  | MUC5B (30) | The increase of mucin is considered to be associated with many kinds of adenocarcinoma, and MUC5B is significantly overexpressed in epithelial cell population | Oncogene |
|  | TM4SF1 (31) | A cell surface antigen and is highly expressed in different carcinomas | Oncogene |
|  | REG4 (32) | May be involved in inflammatory and metaplastic responses of the gastrointestinal epithelium | Oncogene |
|  | TNFRSF6B (38) | A trend association between TNFRSF6B SNP with Crohn's disease susceptibility |  |
|  | TFF1 (33) | Stabilizer of the mucous gel overlying the gastrointestinal mucosa that provides a physical barrier against various noxious agents | Oncogene |
|  | GDF15 (34) | Bind various TGF-beta receptors leading to recruitment and activation of SMAD family transcription factors that regulate gene expression | Oncogene |
|  | IGHA2 | Gene Ontology (GO) annotations related to this gene include antigen binding and immunoglobulin receptor binding |  |
|  | NOS2 (37) | Acts as a biologic mediator in several processes, including neurotransmission and antimicrobial and antitumoral activities. | Tumor suppressor |
|  | ANXA2 (35) | ANXA2 expression has been found to correlate with resistance to treatment against various cancer forms | Oncogene |
| **Enterocyte progenitor cell subset 0 (Entero0)** | DUOX2 (27) | A member of the NADPH oxidase family, promotes the progression of colorectal cancer cells by regulating the AKT pathway and interacting with RPL3 | Oncogene |
|  | AC007952.4 | Unknown | Unknown |
|  | LCN2 (23) | As an oncogenic immune protein in tumor microenvironment | Oncogene |
|  | SELENBP1 (41) | A member of the selenium-binding protein family. Selenium exhibits potent anticarcinogenic properties maybe associated with SELENBP1 | Tumor suppressor |
|  | LAMB1 (50) | Are the major noncollagenous constituent of basement membranes. They have been implicated in a wide variety of biological processes including cell adhesion, differentiation, migration, signaling, neurite outgrowth and metastasis | Higher expression |
|  | AXIN2 (35) | Presumably plays an important role in the regulation of the stability of beta-catenin in the Wnt signaling pathway | Oncogene |
|  | PI3 (24) | Functions as an antimicrobial peptide against Gram-positive and Gram-negative bacteria, and fungal pathogens |  |
|  | APCDD1 (44) | APCDD1 is directly regulated by the beta-catenin/Tcf complex and that its elevated expression is likely to contribute to colorectal tumorigenesis | Oncogene |
|  | CXCL1 (45) | A secreted growth factor that signals through the G-protein coupled receptor | Oncogene |
|  | NOS2 (37) | Antimicrobial, antitumoral activities | Tumor suppressor |
|  | PLA2G2A (16, 36) | Secretory calcium-dependent phospholipase A2, could reduce colon tumorigenesis independent of Apc-germline mutations | Tumor suppressor |
|  | PROM1 (46,47) | Localizes to membrane protrusions and is often expressed on adult stem cells, where it is thought to function in maintaining stem cell properties by suppressing differentiation | Oncogene |
|  | CFTR (52) | Functions as a chloride channel, controls ion and water secretion and absorption in epithelial tissues | Tumor suppressor |
|  | TPM2 (53) | beta-tropomyosin, a member of the actin filament binding protein family | Tumor suppressor |
|  | ASCL2 (48) | It activates transcription by binding to the E box (5'-CANNTG-3') | Oncogene |
|  | HIST1H2BG | Has broad antibacterial activity. May contribute to the formation of the functional antimicrobial barrier of the colonic epithelium | Unknown |
|  | NEK8 | May play a role in cell cycle progression from G2 to M phase | Unknown |
|  | CCL20 (49, 87) | A family of secreted proteins involved in immunoregulatory and inflammatory processes. | Oncogene |
|  | CHRM3 | Mediates various cellular responses, including inhibition of adenylate cyclase, breakdown of phosphoinositides and modulation of potassium channels through the action of G proteins | Unknown |
|  | JAG2 (51) | Putative Notch ligand involved in the mediation of Notch signaling | Higher expression |
| **Enterocyte progenitor cell subset 5 (Entero5)** | DEFA6 (40) | Antimicrobial, highly expressed in the secretory granules of Paneth cells of the small intestine, promotes the proliferation and migration of colon cancer cells | Oncogene |
|  | CLCA1 (58) | May be involved in the regulation of mucus production and/or secretion by goblet cells. May play a role as a tumor suppressor | Tumor suppressor |
|  | IFI6 (54) | Negatively regulating the intrinsinc apoptotic signaling pathway and TNFSF10-induced apoptosis | Oncogene |
|  | TFF3 (33) | Stable secretory proteins expressed in gastrointestinal mucosa, they may protect the mucosa from insults, stabilize the mucus layer and affect healing of the epithelium | Oncogene |
|  | ANO7 (68) | Involved in calcium-activated chloride channel, is a poor prognosis in prostate cancer | Unknown |
|  | SERPINA1 (11) | Produced by the paneth cells of the gut and can promote the tumor progression of CRC | Oncogene |
|  | MUC2 (9) | Secreted and forms an insoluble mucous barrier that protects the gut lumen. High expression of MUC2 is related to the poor prognosis of colon cancer | Oncogene |
|  | LCN2 (23) | An innate immune protein, might serve as a biomarker for immune infiltration and poor prognosis in cancers | Oncogene |
|  | FCGBP (59) | May be involved in the maintenance of the mucosal structure as a gel-like component of the mucosa | Tumor suppressor |
|  | ST6GALNAC1 (10) | ST6GALNAC1 adds sialic acid to O-linked GalNAc residues. Plays important roles in enhancing cancer stem phenotypes of colorectal cancer | Oncogene |
|  | L1TD1 (60) | Diseases associated with L1TD1 include Testis Seminoma | Tumor suppressor |
|  | REP15 (56) | Interacts with GTP-bound Rab15 and is involved in recycling of transferrin receptor from the endocytic recycling compartment to the cell surface | Oncogene |
|  | ATOH1 (62) | Transcription factor, diseases associated with ATOH1 include goblet cell carcinoid | Tumor suppressor |
|  | B3GNT6 (69) | Plays an important role in mucin type O-glycan biosynthesis | Unknown |
|  | KCNMA1 (64) | Large conductance, voltage and calcium-sensitive potassium channels which are fundamental to the control of smooth muscle tone and neuronal excitability | Lower expression. Unknown |
|  | IGFBP2 (57) | High expression levels of this protein promote the growth of several types of tumors and may be predictive of the chances of recovery of the patient | Oncogene |
|  | NEURL1 | Diseases associated with NEURL1 include medulloblastoma and malignant astrocytoma | Unknown |
|  | WFDC2 (65) | Functions as a protease inhibitor. WFDC2-deficiency remarkably improved the radiation resistance in CRC | Tumor suppressor |
|  | SPNS2 (66,67) | Acts a a crucial lysosphingolipid sphingosine 1-phosphate (S1P) transporter involved in S1P secretion and function | Oncogene or tumor suppressor with oppisite result |
|  | LINC00261 (63) | A long non-coding RNA that acts as a negative regulator of cell growth. May promote differentiation and apoptosis | Tumor suppressor |
| **T cell subset 8 (T8)** | FOXP3 (78) | Plays a suppressive role in the immune system especilly for Treg |  |
|  | CTLA4 (70) | Transmits an inhibitory signal to T cells | Immune checkpoint |
|  | MAF (79) | Preserve the identity and function of intestinal Treg cells |  |
|  | TBC1D4 (81) | A Rab-GTPase-activating protein, play an important role in glucose homeostasis | Functions as an oncogene |
|  | ENTPD1 (82) | The rate-limiting enzyme of a cascade, alters CD4 and CD8 T cell and natural killer cell antitumor activities |  |
|  | PIM2 (77) | Plays a prominent role in suppressing T cell responses |  |
|  | BIRC3 (80) | A deleterious role of the TNFα/TNFR2/BIRC3/TRAF1 signaling cascade in NK cells |  |
|  | TTN (74) | TTN mutation as a predictor of improved outcomes in response to Immune checkpoint blockade (ICB) |  |
|  | NAMPT (75) | High NAMPT-expressing tumors are more sensitive to anti-PD-L1 treatment |  |
|  | CTSC (76) | CTSC activates a number of granule-associated serine proteases with pro-inflammatory and immune functions, is a therapeutic target for the treatment of a number of inflammatory and autoimmune diseases | Therapeutic target |
|  | TIGIT (71) | Is an inhibitory receptor expressed on lymphocytes that was recently propelled under the spotlight as a major emerging target in cancer immunotherapy | Immune checkpoint |
|  | ARPC1B (83) | ARPC1B is indispensable for the maintenance of TCR, CD8, and GLUT1 membrane proteins at the plasma membrane of CTLs |  |
|  | LTB (84) | LTB anchors lymphotoxin-alpha to the cell surface through heterotrimer formation |  |
|  | TNFRSF1B (84) | Function after initial T cell activation to sustain T cell responses |  |
|  | C2CD4A | May be involved in inflammatory process and may regulate cell architecture and adhesion |  |
|  | GBP4 (73) | Might be as potential novel immune checkpoint genes of colorectal cancer |  |
|  | CXCR6 (86) | CXCR6 inhibits hepatocarcinogenesis by promoting Natural Killer T- and CD4 + T-Cell-Dependent control of senescence |  |
|  | CCL20 (49, 87) | CCR6 and CCL20 are partners in intestinal immunity and lymph organogenesis |  |
|  | GBP5 | GBP5 also acts as an activator of NLRP3 inflammasome assembly and has a role in innate immunity and inflammation |  |
|  | ICOS (72) | ICOS activation might potentiate the effect of an inhibitory checkpoint blockade, while its neutralization could decrease the function of immunosuppressive Tregs | Immune checkpoint |
